# An new elastic–plastic analytical solution of circular tunnel under non-axisymmetric conditions

**DOI:** 10.1038/s41598-022-08353-3

**Published:** 2022-03-14

**Authors:** Guofeng Li, Ning Li, Yue Bai, Min Yang

**Affiliations:** 1 Institute of Geotechnical Engineering, Xi’an University of Technology, Xi’an, 710048 China; 2 Top International Engineering Corporation, Xi’an, 710054 China

**Keywords:** Engineering, Physics

## Abstract

The surrounding rock is in the initial stress state before the tunnel excavation, and it undergoes the stress redistribution to reach the secondary stress state after the tunnel excavation. The surrounding rock is not only the main load source but also is an important part of the load bearing structure. Once the stress of some zone in the surrounding rock exceeds the strength of the rock mass, the part surrounding rock will enter into a state of plasticity or failure. Analytical solution is the most powerful mean to analyze this kind of underground cavern engineering problems. However, the existing solutions can not directly or simultaneously obtain the secondary stress field and excavation disturbance displacement field that we are concerned about. The implicit approximate solution cannot be degenerated to an accurate axisymmetric expression for the most existing non-axisymmetric elastic–plastic solutions. Therefore, this paper derives the elastic solution of a circular tunnel under the condition of the non-axisymmetric external load with the radial and shear inner loads. On this basis, the new elastic–plastic solution and the plastic zone radius equation of the circular tunnel under the conditions of the non-axisymmetric external load with radial inner load are derived. It can directly obtain the secondary stress field and excavation disturbance displacement field, and can degenerate to an accurate axisymmetric expression. The distribution characteristics of the analytical solution are in good agreement with the numerical test results, indicating that the new solution can provide a relatively accurate theoretical basis for the analysis and research of tunnel engineering.

## Introduction

The surrounding rock is in the initial stress state before the tunnel excavation, and a certain range surrounding rock around the tunnel undergoes stress redistribution to reach the secondary stress state after the tunnel excavation. Li et al.^1^ pointed out that the surrounding rock is not only the main load source but also is an important part of the load bearing structure. Combining the basic mechanic equations and the specific engineering boundary conditions to find more accurate analytical solutions, is the most powerful mean to analyze this type of underground cavern engineering problems, and also has more general significance. The analytical solution can also provide accurate theoretical basis to be used for the analysis and design of tunnel engineering. The existing non-axisymmetric elastic–plastic solutions of Yu et al.^2^ are mostly implicit approximate solutions, which cannot be degenerated to an accurate axisymmetric expression. Meanwhile, the secondary stress field and excavation disturbance displacement field that we are concerned about cannot be obtained directly.

There are three kinds classical elastic analytical solution of circular tunnel, which include the Lame solution of Fairhurst et al.^3^ based on the thick wall barrel theory, the Kirsh solution of Timoshenko et al.^4^ based on the hole orifice problem, and the superposition solution of Li et al.^1^ based on the actual construction process. The comprehensive solution on the linear elastic plane strain problem of a circular tunnel is obtained by Pender^5^, but the deformation field of the surrounding rock did not consider the influence from the deformation of the initial stress field. Goodman^6^ used Kirsh equation to solve the stress and the displacement field distribution on the surrounding rock of the circular tunnel under the bidirectional load. The displacement field distribution removed the influence of the initial in-situ stress, but the stress field did not superimpose the initial stress field. Lv^7–9^ also derived the elastic stress and displacement solution of the circular tunnel by the complex variable function method. However, there are few reports on the elastic analytical solution of circular under the condition of non-axisymmetric external load with the radial and shear internal load.

The elastic–plastic analytical solution put forward by Tarob^10^ on the tunnel are started from Castner's equation and modified Fenner's equation. The assumptions and the results of the two equations are the same. Only the forms of the expression are different. Daemen^11^, Brown et al.^12^, Park et al.^13^, Fan et al.^14^, Xia et al.^15^, Gao et al.^16^, Pronina^17^, Zhang^18^, Zareifard et al.^19^, Masoudian et al.^20^, Zhang et al.^21^, Kargar^22^, Kabwe et al.^23^, Zaheri et al.^24,25^ and Li et al.^26^ and so on, deeply researched or detailedly summarized the elasto-plastic analytical solution of tunnel under axisymmetric conditions. Regarding the elastic–plastic analytical solution of a tunnel under the non-axisymmetric conditions, domestic and foreign researchers, for example, Daemen^11^, Brown et al.^12^, Xu et al.^27^ and so on, have also conducted a lot of research. Kastner^28^ gave an implicit approximate solution for the plastic zone of surrounding rock under non-axisymmetric load conditions. Savin^29,30^ aimed at the plane strain elastic–plastic problem on the circular tunnel, considering the conditions of the inner normal pressure and the non-hydrostatic pressure load at infinity. But Wen et al.^31^ pointed that the solution only can be solved when the lateral pressure coefficient need to satisfy certain conditions. Yu^2^ proposed two approximate calculation methods for the plastic zone stress of the surrounding rock, and revised Kastner's solution to obtain the elastic–plastic boundary equation of the circular tunnel. Though the solution is amended, the elastic–plastic zone boundary cannot be degenerated into a modified Fenner or Castner axisymmetric plastic zone radius equation. Wen et al.^31^ also obtained the elastic–plastic stress and elastic–plastic zone boundary equations of the surrounding rock of the circular tunnel under non-axisymmetric load conditions via the perturbation method. Then Cai et al.^32^ carried out the displacement distribution of the elastic–plastic zone of the surrounding rock of the tunnel under non-axisymmetric load. However, the above solutions are mostly based on certain assumptions, either implicit functions or incomplete solutions, which have certain limitations in the application.

For the tunnel engineering, we are concerned about the secondary stress field and the excavation disturbance displacement field. The research results of Liu et al.^33^ and He et al.^34^ show that once the stress of some areas in the surrounding rock exceeds the strength of the rock mass, the surrounding rock will enter a state of plasticity or failure. Although the elastic and elastic–plastic analytical solution on the surrounding rock of the circular tunnel has made considerable progress, there are still two defects: the displacement solution includes the initial displacement generated by the initial stress field, and the stress solution is only the disturbed stress caused by excavation and does not superimpose the initial stress. Meanwhile, the existing non-axisymmetric elastic–plastic solutions are mostly approximate implicit solution. It cannot be simplified to accurate axisymmetric expressions. Therefore, this paper derives the elastic analytical solution of a circular tunnel under the condition of the non-axisymmetric external loads with the radial and the shear inner load based on the thick wall barrel theory. On this basis, the new elastic–plastic solution and the plastic zone radius equation of the circular tunnel under the conditions of the non-axisymmetric external load with radial inner load are derived. And the analytical solution is verified and discussed by numerical solution test.

## New elastic analytical solution

**Figure 1:**
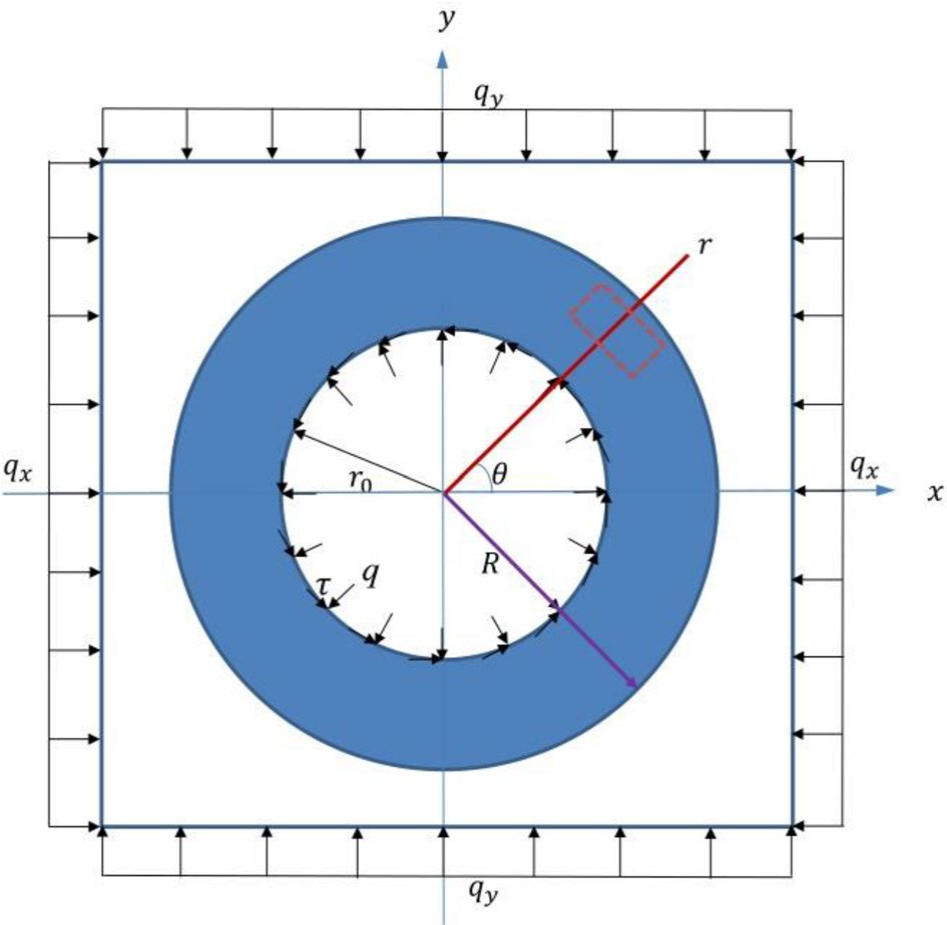
**T**hick wall barrel theory model.

For the elastic analytical solution of the circular tunnel under non-axisymmetric load, there are the derived faults of the expression equation or the printing errors in book of Yu et al.^2^. It is necessary to obtain the accurate elastic analytical solution of circular tunnel again under the condition of the non-axisymmetric external load with the radial and the shear inner loads based on the thick wall barrel theory, in order to solve the elastic–plastic analytical solution under the condition of the non-axisymmetric external load with the radial inner loads. It is assumed that the surrounding rock of a deep buried tunnel is a continuous homogeneous and isotropic medium. The inner radius is ${r}_{0}$, the outer radius is $R$, as shown in Fig. 1, the radial inner load is $q$, the shear is $\tau$, the non-axisymmetric external load are ${q}_{x}$ and ${q}_{y}$ in the $x$ and $y$ directions, respectively. And $E$ and $\mu$ are the deformation modulus and the poisson's ratio of the surrounding rock mass, respectively.

### Compatibility Equations

The stress compatibility equation under non-axisymmetric conditions is,

1 $${\left(\frac{{\partial }^{2}}{\partial {r}^{2}}+\frac{1}{r}\frac{\partial }{\partial r}+\frac{1}{{r}^{2}}\frac{{\partial }^{2}}{\partial {\theta }^{2}}\right)}^{2}\psi =0$$

Constructing a function, $\psi \left(r,\theta \right)={f}_{1}\left(r\right)+{f}_{2}\left(r\right)\mathit{cos}2\theta$, then the above Eq. (1) can be simplified to,

2 $$\frac{{d}^{4}{f}_{1}\left(r\right)}{d{r}^{4}}+\frac{2}{r}\frac{{d}^{3}{f}_{1}\left(r\right)}{d{r}^{3}}+\frac{-1}{{r}^{2}}\frac{{d}^{2}{f}_{1}\left(r\right)}{d{r}^{2}}+\frac{1}{{r}^{3}}\frac{d{f}_{1}\left(r\right)}{dr}+\left(\frac{{d}^{4}{f}_{2}\left(r\right)}{d{r}^{4}}+\frac{2}{r}\frac{{d}^{3}{f}_{2}\left(r\right)}{d{r}^{3}}+\frac{-9}{{r}^{2}}\frac{{d}^{2}{f}_{2}\left(r\right)}{d{r}^{2}}+\frac{9}{{r}^{3}}\frac{d{f}_{2}\left(r\right)}{dr}\right)\mathit{cos}2\theta =0$$

In order to satisfy the Eq. (2) for any θ, the above equation can be separated into two Euler Eqs. (3) and (4),

3 $$\frac{{d}^{4}{f}_{1}\left(r\right)}{d{r}^{4}}+\frac{2}{r}\frac{{d}^{3}{f}_{1}\left(r\right)}{d{r}^{3}}+\frac{-1}{{r}^{2}}\frac{{d}^{2}{f}_{1}\left(r\right)}{d{r}^{2}}+\frac{1}{{r}^{3}}\frac{d{f}_{1}\left(r\right)}{dr}=0$$

4 $$\frac{{d}^{4}{f}_{2}\left(r\right)}{d{r}^{4}}+\frac{2}{r}\frac{{d}^{3}{f}_{2}\left(r\right)}{d{r}^{3}}+\frac{-9}{{r}^{2}}\frac{{d}^{2}{f}_{2}\left(r\right)}{d{r}^{2}}+\frac{9}{{r}^{3}}\frac{d{f}_{2}\left(r\right)}{dr}=0$$

Introducing an intermediate variable,$r={e}^{t}$, then

5 $$\frac{{d}^{4}{f}_{1}\left(t\right)}{d{t}^{4}}-4\frac{{d}^{3}{f}_{1}\left(t\right)}{d{t}^{3}}+4\frac{{d}^{2}{f}_{1}\left(t\right)}{d{t}^{2}}=0$$

Equation (5) can be solved as

6 $${f}_{1}\left(t\right)=At+Bt{e}^{2t}+C{e}^{2t}+D$$

Since, $$t=lnr$$, then

7 $${f}_{1}\left(r\right)={A}_{1}lnr+{B}_{1}{r}^{2}lnr+{C}_{1}{r}^{2}+{D}_{1}$$

In the same way, we can get

8 $${f}_{2}\left(r\right)={A}_{2}{r}^{4}+{B}_{2}{r}^{2}+{C}_{2}+\frac{{D}_{2}}{{r}^{2}}$$

Then the stress function can be expressed as,

9 $$\psi \left(r,\theta \right)={A}_{1}lnr+{B}_{1}{r}^{2}lnr+{C}_{1}{r}^{2}+{D}_{1}+\left({A}_{2}{r}^{4}+{B}_{2}{r}^{2}+{C}_{2}+\frac{{D}_{2}}{{r}^{2}}\right)\mathit{cos}2\theta $$

According to the above stress compatibility Eq. (9), each stress component can be obtained directly. The displacement component can be obtained by bringing the stress component into the physical equation and geometric equation, and then by integrating according to the single value condition and the symmetry condition of the displacement. The simplified stress component and displacement component can be obtained as shown below expression.

10 $${\sigma }_{r}=\left(\frac{{A}_{1}}{{r}^{2}}+{2C}_{1}\right)+\left(-\frac{6{D}_{2}}{{r}^{4}}-2{B}_{2}-4\frac{{C}_{2}}{{r}^{2}}\right)\mathit{cos}2\theta $$

11 $${\sigma }_{\theta }=\left(-\frac{{A}_{1}}{{r}^{2}}+{2C}_{1}\right)+\left({12A}_{2}{r}^{2}+{2B}_{2}+\frac{6{D}_{2}}{{r}^{4}}\right)\mathit{cos}2\theta $$

12 $${\tau }_{r\theta }=2\left({3A}_{2}{r}^{2}+{B}_{2}-\frac{{3D}_{2}}{{r}^{4}}-\frac{{C}_{2}}{{r}^{2}}\right)\mathit{sin}2\theta $$

13 $${u}_{r}E=\left[-\frac{{A}_{1}}{r}\left(1+\mu \right)+{2C}_{1}\left(1-\mu \right)r\right]+\left[\frac{2{D}_{2}}{{r}^{3}}\left(1+\mu \right)-2{B}_{2}\left(1+\mu \right)r+4\frac{{C}_{2}}{r}{-4A}_{2}{r}^{3}\mu \right]\mathit{cos}2\theta $$

14 $${u}_{\theta }E =\left[2\left(1+\mu \right)\frac{{D}_{2}}{{r}^{3}}+{2B}_{2}\left(1+\mu \right)r+{\left(6+2\mu \right)A}_{2}{r}^{3}+2\frac{{C}_{2}}{r}\left(\mu -1\right)\right]\mathit{sin}2\theta $$

### Coefficient

Outer stress boundary conditions,

15 $${\left({\sigma }_{r}\right)}_{r=R}=\frac{{q}_{x}+{q}_{y}}{2}+\frac{{q}_{x}-{q}_{y}}{2}\mathit{cos}2\theta =\left[\frac{{A}_{1}}{{R}^{2}}+{2C}_{1}\right]+\left[-\frac{6{D}_{2}}{{R}^{4}}-2{B}_{2}-4\frac{{C}_{2}}{{R}^{2}}\right]\mathit{cos}2\theta $$

16 $${\left({\tau }_{r\theta }\right)}_{r=R}=\frac{{q}_{y}-{q}_{x}}{2}\mathit{sin}2\theta =2\left({3A}_{2}{R}^{2}+{B}_{2}+\frac{{-3D}_{2}}{{R}^{4}}-\frac{{C}_{2}}{{R}^{2}}\right)\mathit{sin}2\theta $$

Inner stress boundary conditions,

17 $${\left({\sigma }_{r}\right)}_{r={r}_{0}}=q=\left(\frac{{A}_{1}}{{r}_{0}^{2}}+{2C}_{1}\right)+\left(-\frac{6{D}_{2}}{{r}_{0}^{4}}-2{B}_{2}-4\frac{{C}_{2}}{{r}_{0}^{2}}\right)\mathit{cos}2\theta $$

18 $${\left({\tau }_{r\theta }\right)}_{r={r}_{0}}=\tau =2\left({3A}_{2}{r}_{0}^{2}+{B}_{2}+\frac{{-3D}_{2}}{{r}_{0}^{4}}-\frac{{C}_{2}}{{r}_{0}^{2}}\right)\mathit{sin}2\theta \ne 0$$

Note that, there is no circumference stress ${\sigma }_{\theta }$ boundary condition for the free surface, and there are only radial stress ${\sigma }_{r}$ and shear stress ${\tau }_{r\theta }$ on the boundary.

When the outer radius of the tunnel tends to infinity,$R\to \infty$, the following coefficients can be obtained.

19 $${A}_{1}=q{r}_{0}^{2}-\frac{{q}_{x}+{q}_{y}}{2}{r}_{0}^{2}$$

20 $${C}_{1}=\frac{{q}_{x}+{q}_{y}}{4}$$

21 $${A}_{2}=0$$

22 $${B}_{2}=-\frac{{q}_{x}-{q}_{y}}{4}$$

23 $${C}_{2}={r}_{0}^{2}\left(\frac{\tau }{2\mathit{sin}2\theta }+\frac{{q}_{x}-{q}_{y}}{2}\right)$$

24 $${D}_{2}=-{r}_{0}^{4}\left(\frac{\tau }{3\mathit{sin}2\theta }+\frac{{q}_{x}-{q}_{y}}{4}\right)$$

### Stress and displacement components

Substituting the coefficients Eqs. (19–24) into the stress and displacement component Eqs. (10–14), the stress and displacement components of the circular tunnel under non-axisymmetric conditions can be obtained.

25 $${\sigma }_{r}=\left(1-\frac{{r}_{0}^{2}}{{r}^{2}}\right)\frac{{q}_{x}+{q}_{y}}{2}+\left(1+\frac{3{r}_{0}^{4}}{{r}^{4}}-\frac{4{r}_{0}^{2}}{{r}^{2}}\right)\frac{{q}_{x}-{q}_{y}}{2}\mathit{cos}2\theta +\frac{{r}_{0}^{2}}{{r}^{2}}q+\left(\frac{{r}_{0}^{4}}{{r}^{4}}-\frac{{r}_{0}^{2}}{{r}^{2}}\right)2\tau \mathit{cot}2\theta $$

26 $${\sigma }_{\theta }=\left(1+\frac{{r}_{0}^{2}}{{r}^{2}}\right)\frac{{q}_{x}+{q}_{y}}{2}-\left(1+\frac{3{r}_{0}^{4}}{{r}^{4}}\right)\frac{{q}_{x}-{q}_{y}}{2}\mathit{cos}2\theta -\frac{{r}_{0}^{2}}{{r}^{2}}q-2\tau \frac{{r}_{0}^{4}}{{r}^{4}}\mathit{cot}2\theta $$

27 $${\tau }_{r\theta }=-\left(1+\frac{2{r}_{0}^{2}}{{r}^{2}}-\frac{3{r}_{0}^{4}}{{r}^{4}}\right)\frac{{q}_{x}-{q}_{y}}{2}\mathit{sin}2\theta +\left(2\frac{{r}_{0}^{4}}{{r}^{4}}-\frac{{r}_{0}^{2}}{{r}^{2}}\right)\tau $$

28 $$ \begin{aligned}{u}_{r}E&=\left[\left(\frac{{r}_{0}^{2}}{r}\left(1+\mu \right)+\left(1-\mu \right)r\right)\frac{{q}_{x}+{q}_{y}}{2}\right]+\left[\left(\left(r-\frac{{r}_{0}^{4}}{{r}^{3}}\right)\left(1+\mu \right)+4\frac{{r}_{0}^{2}}{r}\right)\frac{{q}_{x}-{q}_{y}}{2}\right]\mathit{cos}2\theta \\ &\quad-\frac{{r}_{0}^{2}}{r}q\left(1+\mu \right)+2\tau \left(\frac{{r}_{0}^{2}}{r}-\frac{\left(1+\mu \right){r}_{0}^{4}}{3{r}^{3}}\right)\mathit{cot}2\theta \end{aligned} $$

29 $${u}_{\theta }E =\left[2\frac{{r}_{0}^{2}}{r}\left(\mu -1\right)-\left(1+\mu \right)\left(\frac{{r}_{0}^{4}}{{r}^{3}}+r\right)\right]\frac{{q}_{x}-{q}_{y}}{2}\mathit{sin}2\theta +\left[\frac{{r}_{0}^{2}}{r}\left(\mu -1\right)-\frac{2}{3}\frac{{r}_{0}^{4}}{{r}^{3}}\left(1+\mu \right)\right]\tau $$

When the inter radius of the tunnel tends to zero, ${r}_{0}\to 0$, the displacement generated by the initial stress field can be obtained,

30 $${u}_{r}E=\frac{{q}_{x}+{q}_{y}}{2}\left(1-\mu \right)r+\frac{{q}_{x}-{q}_{y}}{2}\left(1+\mu \right)r\mathit{cos}2\theta $$

31 $${u}_{\theta }E =-\frac{{q}_{x}-{q}_{y}}{2}\left(1+\mu \right)r\mathit{sin}2\theta $$

The excavation disturbance displacement is the difference of the displacement produced by the initial stress field ${r}_{0}\to 0$, and the displacement produced by the inner radius ${r}_{0}$,

32 $${u}_{r}E=\left[\frac{{r}_{0}^{2}}{r}\left(1+\mu \right)\right]\frac{{q}_{x}+{q}_{y}}{2}+\left[-\left(1+\mu \right)\frac{{r}_{0}^{4}}{{r}^{3}}+4\frac{{r}_{0}^{2}}{r}\right]\frac{{q}_{x}-{q}_{y}}{2}\mathit{cos}2\theta -\frac{{r}_{0}^{2}}{r}q\left(1+\mu \right)+2\tau \left[\frac{{r}_{0}^{2}}{r}-\frac{\left(1+\mu \right){r}_{0}^{4}}{3{r}^{3}}\right]\mathit{cot}2\theta $$

33 $${u}_{\theta }E =\left[\frac{2{r}_{0}^{2}}{r}\left(\mu -1\right)-\left(1+\mu \right)\frac{{r}_{0}^{4}}{{r}^{3}}\right]\frac{{q}_{x}-{q}_{y}}{2}\mathit{sin}2\theta +\left[\frac{{r}_{0}^{2}}{r}\left(\mu -1\right)-\frac{2}{3}\left(1+\mu \right)\frac{{r}_{0}^{4}}{{r}^{3}}\right]\tau $$

For the plane strain problem, just replace $E$ with $\frac{E}{1-{\mu }^{2}}$ in the above equations, and replace $\mu$ with $\frac{\mu }{1-\mu }$.

34 $${u}_{r}=\frac{1+\mu }{E}\left\{\left(\frac{{r}_{0}^{2}}{r}\right)\frac{{q}_{x}+{q}_{y}}{2}+\left[-\frac{{r}_{0}^{4}}{{r}^{3}}+\left(1-\mu \right)4\frac{{r}_{0}^{2}}{r}\right]\frac{{q}_{x}-{q}_{y}}{2}\mathit{cos}2\theta -\frac{{r}_{0}^{2}}{r}q+2\tau \left[\frac{{r}_{0}^{2}}{r}\left(1-\mu \right)-\frac{{r}_{0}^{4}}{3{r}^{3}}\right]\mathit{cot}2\theta \right\}$$

35 $${u}_{\theta } =\frac{1+\mu }{E}\left\{\left[\frac{2{r}_{0}^{2}}{r}\left(2\mu -1\right)-\frac{{r}_{0}^{4}}{{r}^{3}}\right]\frac{{q}_{x}-{q}_{y}}{2}\mathit{sin}2\theta +\left[\frac{{r}_{0}^{2}}{r}\left(2\mu -1\right)-\frac{2}{3}\frac{{r}_{0}^{4}}{{r}^{3}}\right]\tau \right\}$$

When $\lambda ={q}_{x}/{q}_{y}$ and $P={q}_{y}$, the stress and displacement components of a circular tunnel under non-axisymmetric conditions are as follow,

36 $${\sigma }_{r}=\frac{P}{2}\left(\lambda +1\right)\left(1-\frac{{r}_{0}^{2}}{{r}^{2}}\right)+\frac{P}{2}\left(\lambda -1\right)\left(1+\frac{3{r}_{0}^{4}}{{r}^{4}}-\frac{4{r}_{0}^{2}}{{r}^{2}}\right)\mathit{cos}2\theta +\frac{{r}_{0}^{2}}{{r}^{2}}q+2\tau \left(\frac{{r}_{0}^{4}}{{r}^{4}}-\frac{{r}_{0}^{2}}{{r}^{2}}\right)\mathit{cot}2\theta $$

37 $${\sigma }_{\theta }=\frac{P}{2}\left(\lambda +1\right)\left(1+\frac{{r}_{0}^{2}}{{r}^{2}}\right)-\frac{P}{2}\left(\lambda -1\right)\left(1+\frac{3{r}_{0}^{4}}{{r}^{4}}\right)\mathit{cos}2\theta -\frac{{r}_{0}^{2}}{{r}^{2}}q-2\tau \frac{{r}_{0}^{4}}{{r}^{4}}\mathit{cot}2\theta $$

38 $${\tau }_{r\theta }=-\frac{P}{2}\left(\lambda -1\right)\left(1+\frac{2{r}_{0}^{2}}{{r}^{2}}-\frac{3{r}_{0}^{4}}{{r}^{4}}\right)\mathit{sin}2\theta +\left(2\frac{{r}_{0}^{4}}{{r}^{4}}-\frac{{r}_{0}^{2}}{{r}^{2}}\right)\tau $$

39 $${u}_{r}=\frac{1+\mu }{E}\left\{\left(\frac{{r}_{0}^{2}}{r}\right)\frac{P}{2}\left(\lambda +1\right)+\left[-\frac{{r}_{0}^{4}}{{r}^{3}}+\left(1-\mu \right)4\frac{{r}_{0}^{2}}{r}\right]\frac{P}{2}\left(\lambda -1\right)\mathit{cos}2\theta -\frac{{r}_{0}^{2}}{r}q+2\tau \left[\frac{{r}_{0}^{2}}{r}\left(1-\mu \right)-\frac{{r}_{0}^{4}}{3{r}^{3}}\right]\mathit{cot}2\theta \right\}$$

40 $${u}_{\theta } =\frac{1+\mu }{E}\left\{\left[\frac{2{r}_{0}^{2}}{r}\left(2\mu -1\right)-\frac{{r}_{0}^{4}}{{r}^{3}}\right]\frac{P}{2}\left(\lambda -1\right)\mathit{sin}2\theta +\left[\frac{{r}_{0}^{2}}{r}\left(2\mu -1\right)-\frac{2}{3}\frac{{r}_{0}^{4}}{{r}^{3}}\right]\tau \right\}$$

The above equation can be degenerated into Xu’ elastic solution^35^ under the condition of non-axisymmetric external load without internal load, when the internal load $q=0$ and $\tau =0$.

## New elastic–plastic analytical solution

The stress and deformation of surrounding rock are functions of $r$ and have nothing to do with $\theta$ under the axisymmetric conditions. The basic principle of solving the elastic–plastic solution is that the plastic zone satisfies the plastic condition and the plastic balance equation, the elastic zone satisfies the elastic condition and the elastic balance equation, and the elastic–plastic interface satisfies both the elastic condition and the plastic condition. However, for the non-axisymmetric conditions, the stress and deformation of surrounding rock are functions of $r$ and $\theta$, and the plastic zone solution is difficult to solve accurately. Generally, only the implicit expression of the radius of the plastic zone can be obtained by bringing the stress solution of the surrounding rock according to the elastic theory into the plastic condition. And the plastic zone can be approximated by connecting the points satisfying the plastic condition.

For the Yu’ solution^2^ based on the assumption of axisymmetric stress distribution, the plastic zone solution does not completely satisfy the plastic yield condition, so there is a certain error and it is not satisfied the axisymmetric condition. The expression of the yield function under the no-axisymmetric condition in the Kastner solution is correct, but implicit function of the plastic zone radius is incorrectly deduced.

In this section, the basic idea of deriving the elasto-plastic solution of a circular tunnel under the condition of the non-axisymmetric external load with radial inner load is as follows,

1. A relatively accurate stress solution of the plastic zone is obtained based on the assumption of stress distribution in the elastic–plastic zone from Yu et al.^2^ and the plastic conditions. The assumption is that the radial stress expressions of the elastic and plastic zone are the same, and the principal stress directions are the same.
2. The plastic zone radius equation under non-axisymmetric conditions is derived according to the elastic–plastic boundary stress coordination condition, ${\sigma }_{r}^{e}+{\sigma }_{\theta }^{e}={\sigma }_{r}^{p}+{\sigma }_{\theta }^{p}$.
3. The stress and displacement solution of the elastic zone considering the action of the plastic zone are derived based on the elastic–plastic boundary stress conditions, ${\sigma }_{r}^{*}={\sigma }_{r}^{e}={\sigma }_{r}^{p}={P}_{i}$ and ${\tau }_{r\theta }^{*}={\tau }_{r\theta }^{e}={\tau }_{r\theta }^{p}={\tau }_{i}$.
4. The displacement solution of the plastic zone is derived based on the small deformation theory and the elastic–plastic boundary displacement coordination condition,${{u}^{e}=u}^{p}$.

### General form of Mohr–Coulomb yield criterion

If ${\tau }_{n}$ and ${\sigma }_{n}$ are represented by the principal stresses ${\sigma }_{1}$ and ${\sigma }_{3}$, ${\sigma }_{1}\ge {\sigma }_{2}\ge {\sigma }_{3}$, and the compressive stress is positive, it can be obtained from the Mohr circle,

41 $${\tau }_{n}=\mathit{tan}\varphi {\sigma }_{n}+C$$

42 $${\sigma }_{1}-{\sigma }_{3}\frac{1+\mathit{sin}\varphi }{1-\mathit{sin}\varphi }=\frac{2\mathit{Ccos}\varphi }{1-\mathit{sin}\varphi }$$

43 $${\sigma }_{1}=\xi {\sigma }_{3}+{R}_{c}$$

where, ${\sigma }_{1}$ is the max principal stress, ${\sigma }_{3}$ is the min principal stress, ${R}_{c}$ is the theoretical uniaxial compressive strength, ${R}_{c}=\frac{2c\mathrm{cos}\varphi }{1-\mathrm{sin}\varphi },\xi$ is the rate of the intensity line, $\xi =\frac{1+\mathrm{sin}\varphi }{1-\mathrm{sin}\varphi }$.

According to the stress characteristics of the tunnel, the circumference stress ${\sigma }_{\theta }={\sigma }_{1}$ and the radial stress ${\sigma }_{r}={\sigma }_{3}$ can be approximated, so the plastic condition still can be expressed as,

44 $${\sigma }_{\theta }=\xi {\sigma }_{r}+{R}_{c}$$

### Stress of plastic zone

(1) According to the Eq. (36), if there is only the radial internal load, the radial stress of the non-axisymmetric elastic solution expression is applied as,

45 $${\sigma }_{r}^{e}=\frac{1}{2}P\left[\left(1+\lambda \right)\left(1-{\alpha }^{2}\right)-\left(1-\lambda \right)\left(1+3{\alpha }^{4}-4{\alpha }^{2}\right)\mathit{cos}2\theta +\frac{2{P}_{n}}{P}{\alpha }^{2}\right]$$

where, $\alpha =\frac{{r}_{0}}{r}$, ${P}_{n}$ is the radial internal load.

(2) When $\lambda =1$, if there is only the radial internal load, the radial stress of the axisymmetric elastic solution expression can be obtained as,

46 $${\sigma }_{r}^{e}=P\left[\left(1-{\alpha }^{2}\right)+\frac{{P}_{n}}{P}{\alpha }^{2}\right]$$

(3) According to the modified Fenner equation solution method of Xia et al.^36^, if there is only the radial internal load, the radial stress of the axisymmetric plastic zone solution expression can be obtained as,

47 $${\sigma }_{r}^{p}=\left({P}_{n}+\frac{{R}_{c}}{\xi -1}\right){\left(\frac{r}{{r}_{0}}\right)}^{\xi -1}-\frac{{R}_{c}}{\xi -1}$$

(4) It is assumed that the radial stress expressions of the elastic and plastic zone are the same, and the principal stress directions are the same, which is agree with the assumption of Yu et al.^2^. Then the radial stress expression of the plastic zone under the non-axisymmetric outer boundary conditions with a radial internal load is as follows,

48 $${\sigma }_{r}^{p}=\frac{1}{2}P\left[\left(1+\lambda \right)\left(1-{\alpha }^{2}\right)-\left(1-\lambda \right)\left(1+3{\alpha }^{4}-4{\alpha }^{2}\right)\mathit{cos}2\theta +\frac{2{P}_{n}}{P}{\alpha }^{2}\right]$$

(5) When $\uplambda =1$, according to Eq. (48), the correction radial stress solution of the plastic zone is,

49 $${\sigma }_{r}^{p}=P\left(1-{\alpha }^{2}\right)+{P}_{n}{\alpha }^{2}$$

When $\alpha =\frac{{r}_{0}}{r}$, Eq. (47) can be transformed into the following form,

50 $${\sigma }_{r}^{p}=\left[\frac{{R}_{c}}{\xi -1}{\alpha }^{1-\upxi }\right]\left(1-{\alpha }^{\upxi -1}\right)+\left[{P}_{n}{\alpha }^{2-2\upxi }\right]{\alpha }^{\upxi -1}$$

The above two equations both represent the radial stress of the plastic zone when there is a radial internal load, so they are completely the same form. That is, if $P\to \frac{{R}_{c}}{\xi -1}{\alpha }^{1-\upxi },{\alpha }^{2}\to {\alpha }^{\upxi -1},{P}_{n}\to {P}_{n}{\alpha }^{2-2\upxi }$, Eq. (50) can be obtained. Therefore, by replacing $P,{\alpha }^{2},{P}_{n}$ in Eq. (48), the expression of the radial stress of the plastic zone under the non-axisymmetric condition with the internal load is as follows,

51 $${\sigma }_{r}^{p}=\frac{1}{2}\frac{{R}_{c}}{\xi -1}{\alpha }^{1-\upxi }\left[\left(1+\lambda \right)\left(1-{\alpha }^{\upxi -1}\right)-\left(1-\lambda \right)\left(1+3{\alpha }^{2\upxi -2}-4{\alpha }^{\upxi -1}\right)\mathit{cos}2\theta +\left(\xi -1\right)\frac{2{P}_{n}}{{R}_{c}}\right]$$

(6)The circumference stress expression under the non-axisymmetric condition can be obtained by substituting the radial stress expression Eq. (51) into the plastic condition Eq. (44).

52 $${\sigma }_{\theta }=\frac{\xi }{2}\frac{{R}_{c}}{\xi -1}{\alpha }^{1-\upxi }\left[\left(1+\lambda \right)\left(1-{\alpha }^{\upxi -1}\right)-\left(1-\lambda \right)\left(1+3{\alpha }^{2\upxi -2}-4{\alpha }^{\upxi -1}\right)\mathit{cos}2\theta +\left(\xi -1\right)\frac{2{P}_{n}}{{R}_{c}}\right]+{R}_{c}$$

When $\uplambda =1$, the above equation can be expressed as,

53 $${\sigma }_{\theta }=\left({P}_{n}+\frac{{R}_{c}}{\xi -1}\right)\xi {\alpha }^{1-\upxi }-\frac{{R}_{c}}{\xi -1}$$

The above Eq. (53) is consistent with the exact solution of axisymmetric conditions of Xia et al.^36^. At this time, the radial stress and circumference stress of the plastic zone under non-axisymmetric conditions have been obtained.

(7) According to the Mohr circle characteristic expression Eq. (54) of Yu et al.^2^, the shear stress in the plastic zone can be expressed as Eq. (55).

54 $${\tau }_{r\theta }^{p}=\frac{{\sigma }_{\theta }^{p}-{\sigma }_{r}^{p}}{2}\mathit{tan}2\theta $$

55 $${\tau }_{r\theta }^{p}=\frac{1}{4}\left[{2P}_{n}\left(\xi -1\right)+{R}_{c}\left(1+\lambda \right)\right]{\alpha }^{1-\upxi }\mathit{tan}2\theta +\frac{{R}_{c}}{4}\left(1-\lambda \right)\mathit{tan}2\theta -\frac{{R}_{c}}{4}{\alpha }^{1-\upxi }\left(1-\lambda \right)\left(1+3{\alpha }^{2\upxi -2}-4{\alpha }^{\upxi -1}\right)\mathrm{sin}2\theta $$

When $\uplambda =1$, the above equation can be simplified to,

56 $${\tau }_{r\theta }^{p}=\frac{1}{2}\left[{P}_{n}\left(\xi -1\right)+{R}_{c}\right]{\alpha }^{1-\upxi }\mathit{tan}2\theta \ne 0$$

The above equation is inconsistent with the exact solution under the condition of axisymmetric, so the above equation needs to be corrected axisymmetrically. The modified shear stress expression is,

57 $${\tau }_{r\theta }^{p}=\frac{{R}_{c}}{4}\left(1-\lambda \right)\left[\mathit{tan}2\theta -{\alpha }^{1-\upxi }\mathit{tan}2\theta +\left(4-{\alpha }^{1-\upxi }-3{\alpha }^{\upxi -1}\right)\mathrm{sin}2\theta \right]$$

In summary, the stress component expression of the plastic zone under non-axisymmetric conditions has been obtained as Eqs. (51–52) and (57). In where,$\alpha =\frac{{r}_{0}}{r}$, and $r\le {R}_{*}$, ${R}_{*}$ is the plastic zone radius.

### Radius of plastic zone

(1) Solving the elastic–plastic boundary load.

According to Eqs. (36–38), the elastic zone stress component under the non-axisymmetric condition with internal load can be obtained as,

58 $${\sigma }_{r}^{e}=\frac{1}{2}P\left[\left(1+\uplambda \right)\left(1-{\alpha }^{2}\right)-\left(1-\uplambda \right)\left(1+3{\alpha }^{4}-4{\alpha }^{2}\right)\mathit{cos}2\theta +{\mu }_{0}{\alpha }^{2}+2{\mu }_{0}^{^{\prime}}\left({\alpha }^{4}-{\alpha }^{2}\right)\mathrm{cot}2\theta \right]$$

59 $${\sigma }_{\theta }^{e}=\frac{1}{2}P\left[\left(1+\uplambda \right)\left(1+{\alpha }^{2}\right)+\left(1-\uplambda \right)\left(1+3{\alpha }^{4}\right)\mathit{cos}2\theta -{\mu }_{0}{\alpha }^{2}-2{\mu }_{0}^{^{\prime}}{\alpha }^{4}\mathrm{cot}2\theta \right]$$

60 $${\tau }_{r\theta }^{e}=\frac{1}{2}P\left[\left(1-\uplambda \right)\left(1+{2\alpha }^{2}-3{\alpha }^{4}\right)\mathrm{sin}2\theta +{\mu }_{0}^{^{\prime}}\left({2{\alpha }^{4}-\alpha }^{2}\right)\right]$$

where,$\alpha =\frac{{R}_{*}}{r}, r\ge {R}_{*},{\mu }_{0}=\frac{2{P}_{i}}{P}$,${\mu }_{0}^{^{\prime}}=\frac{2{\tau }_{i}}{P},{P}_{i}$ and ${\tau }_{i}$ are the radial and shear stress of the elastic–plastic boundary, ${\upmu }_{0}$ and ${\upmu }_{0}^{\mathrm{^{\prime}}}$ are intermediate variables, ${R}_{*}$ is the plastic zone radius.

When $r={R}_{*}$, that is, $\alpha =1$, the stress component at the elastic–plastic boundary is,

61 $${\sigma }_{r}^{e}={P}_{i}$$

62 $${\sigma }_{\theta }^{e}=\frac{1}{2}P\left[2\left(1+\uplambda \right)+4\left(1-\uplambda \right)\mathit{cos}2\theta \right]-{P}_{i}-2{\tau }_{i}\mathrm{cot}2\theta $$

63 $${\tau }_{r\theta }^{e}={\tau }_{i}$$

According to the plastic zone expression Eqs. (51–52) and (57), when $r={R}_{*}$, the stress component at the elastic–plastic boundary are,

64 $${P}_{i}=\frac{1}{2}\frac{{R}_{c}}{\xi -1}{\alpha }^{1-\upxi }\left[\left(1+\lambda \right)\left(1-{\alpha }^{\upxi -1}\right)-\left(1-\lambda \right)\left(1+3{\alpha }^{2\upxi -2}-4{\alpha }^{\upxi -1}\right)\mathit{cos}2\theta +\frac{2{P}_{n}}{{R}_{c}}\left(\xi -1\right)\right]$$

65 $${\tau }_{i}=\frac{{R}_{c}}{4}\left(1-\lambda \right)\left[\mathit{tan}2\theta -{\alpha }^{1-\upxi }\mathit{tan}2\theta +\left(4-{\alpha }^{1-\upxi }-3{\alpha }^{\upxi -1}\right)\mathrm{sin}2\theta \right]$$

where, $$\alpha =\frac{{r}_{0}}{{R}_{*}}$$.

(2) Method 1 for solving the plastic zone radius.

According to Eqs. (61–65), the stress component at the elastic–plastic boundary in the elastic zone has an expression as follows,

66 $${\sigma }_{r}^{e}+{\sigma }_{\theta }^{e}=P\left[\left(1+\uplambda \right)+2\left(1-\uplambda \right)\mathit{cos}2\theta \right]-\frac{{R}_{c}}{2}\left(1-\lambda \right)\left[1-{\alpha }^{1-\upxi }+\left(4-{\alpha }^{1-\upxi }-3{\alpha }^{\upxi -1}\right)\mathit{cos}2\theta \right]$$

According to Eqs. (51–52) and (57), the stress component at the elastic–plastic boundary in the plastic zone has an expression as follows,

67 $${\sigma }_{r}^{p}+{\sigma }_{\theta }^{p}=\frac{1}{2}\frac{{R}_{c}}{\xi -1}{\alpha }^{1-\upxi }\left(\xi +1\right)\left[\left(1+\lambda \right)\left(1-{\alpha }^{\upxi -1}\right)-\left(1-\lambda \right)\left(1+3{\alpha }^{2\upxi -2}-4{\alpha }^{\upxi -1}\right)\mathit{cos}2\theta +\frac{2{P}_{n}}{{R}_{c}}\left(\xi -1\right)\right]+{R}_{c}$$

Then there is ${\sigma }_{r}^{e}+{\sigma }_{\theta }^{e}={\sigma }_{r}^{p}+{\sigma }_{\theta }^{p}$, at the elastic–plastic boundary $r={R}_{*}$, that is,

68 $$ \begin{aligned} & P\left[\left(1+\uplambda \right)+2\left(1-\uplambda \right)\mathit{cos}2\theta \right]-\frac{{R}_{c}}{2}\left(1-\lambda \right)\left[1-{\alpha }^{1-\upxi }+\left(4-{\alpha }^{1-\upxi }-3{\alpha }^{\upxi -1}\right)\mathit{cos}2\theta \right]\\ &\quad=\frac{1}{2}\frac{{R}_{c}}{\xi -1}\left(\xi +1\right){\alpha }^{1-\upxi }\left[\left(1+\lambda \right)\left(1-{\alpha }^{\upxi -1}\right)-\left(1-\lambda \right)\left(1+3{\alpha }^{2\upxi -2}-4{\alpha }^{\upxi -1}\right)\mathit{cos}2\theta +\frac{2{P}_{n}}{{R}_{c}}\left(\xi -1\right)\right]+{R}_{c}\end{aligned} $$

The above equation is simplified to the quadratic function of ${\alpha }^{1-\upxi }$ is,

69 $$ \begin{aligned} & \left[{P}_{n}\left(\xi +1\right)-\frac{\xi {R}_{c}}{\xi -1}\left(1-\uplambda \right)\mathit{cos}2\theta -\frac{{R}_{c}}{2}\left(1-\lambda \right)+\frac{{R}_{c}}{2}\frac{\xi +1}{\xi -1}\left(1+\lambda \right)\right]{\alpha }^{2-2\upxi }\\ &\quad-\left[P\left(1+\uplambda \right)-\frac{{R}_{c}}{2}\left(3-\lambda \right)+\frac{{R}_{c}}{2}\frac{\xi +1}{\xi -1}\left(1+\lambda \right)+2P\left(1-\uplambda \right)\mathit{cos}2\theta -4\frac{\xi {R}_{c}}{\xi -1}\left(1-\uplambda \right)\mathit{cos}2\theta \right]{\alpha }^{1-\upxi }\\ &\quad-3\frac{\xi {R}_{c}}{\xi -1}\left(1-\uplambda \right)\mathit{cos}2\theta =0\end{aligned} $$

If

70 $$A=\left[{P}_{n}\left(\xi +1\right)-\frac{\xi {R}_{c}}{\xi -1}\left(1-\uplambda \right)\mathit{cos}2\theta -\frac{{R}_{c}}{2}\left(1-\lambda \right)+\frac{{R}_{c}}{2}\frac{\xi +1}{\xi -1}\left(1+\lambda \right)\right]$$

71 $$B=-\left[P\left(1+\uplambda \right)-\frac{{R}_{c}}{2}\left(3-\lambda \right)+\frac{{R}_{c}}{2}\frac{\xi +1}{\xi -1}\left(1+\lambda \right)+2P\left(1-\uplambda \right)\mathit{cos}2\theta -4\frac{\xi {R}_{c}}{\xi -1}\left(1-\uplambda \right)\mathit{cos}2\theta \right]$$

72 $$C=-3\frac{\xi {R}_{c}}{\xi -1}\left(1-\uplambda \right)\mathit{cos}2\theta $$

The original Eq. (69) can be expressed as,

73 $$A{\alpha }^{2-2\upxi }+B{\alpha }^{1-\upxi }+C=0$$

74 $${\alpha }^{1-\upxi }=\frac{-B+\sqrt{{B}^{2}-4\mathrm{AC}}}{2A}$$

75 $${R}_{*}={r}_{0}{\left[{\alpha }^{1-\upxi }\right]}^{\frac{1}{\upxi -1}}$$

If $\uplambda =1$, the original Eq. (69) can be degenerated into,

76 $${\alpha }^{1-\upxi }=\frac{2}{\xi +1}\frac{P\left(\xi -1\right)+{R}_{c}}{{R}_{c}+{P}_{n}\left(\xi -1\right)}$$

77 $${R}_{*}={r}_{0}{\left[\frac{2}{\xi +1}\frac{P\left(\xi -1\right)+{R}_{c}}{\left(\xi -1\right){P}_{n}+{R}_{c}}\right]}^{\frac{1}{\upxi -1}}$$

The above Eq. (77) is consistent with the exact solution of Xia et al. (2004) under axisymmetric conditions.

(3) Method 2 for solving the plastic zone radius.

When $r={R}_{*}$, according to Eqs. (61) and (62), the stress component at the elastic–plastic boundary in the elastic zone is,

78 $${\sigma }_{r}^{*}+{\sigma }_{\theta }^{*}=\frac{1}{2}P\left[\left(1+\lambda \right)2+\left(1-\lambda \right)4\mathit{cos}2\theta \right]-2{\tau }_{i}\mathit{cot}2\theta $$

When $r={R}_{*}$, according to Eq. (44), the stress component at the elastic–plastic boundary in the plastic zone is,

79 $${\sigma }_{\theta }^{*}=\xi {\sigma }_{r}^{*}+{R}_{c}$$

Combining the above two Eqs. (78) and (79), the stress component at the elastic–plastic boundary can be obtained as,

80 $${\sigma }_{r}^{*}=\frac{1}{\xi +1}\left\{P\left[\left(1+\uplambda \right)+2\left(1-\uplambda \right)\mathit{cos}2\theta \right]-2{\tau }_{i}\mathrm{cot}2\theta -{R}_{c}\right\}$$

81 $${\sigma }_{\theta }^{*}=\frac{1}{\xi +1}\left\{P\left[\left(1+\uplambda \right)+2\left(1-\uplambda \right)\mathit{cos}2\theta \right]-2{\tau }_{i}\mathrm{cot}2\theta -{R}_{c}\right\}\xi +{R}_{c}$$

When $r={R}_{*}$, according to Eq. (54), the shear stress of the plastic zone at the elastic–plastic boundary is,

82 $${\tau }_{r\theta }^{*}={\tau }_{i}=\frac{\mathit{tan}2\theta }{2}\frac{\xi -1}{\xi +1}\left\{P\left[\left(1+\lambda \right)+2\left(1-\lambda \right)\mathit{cos}2\theta \right]-2{\tau }_{i}\mathit{cot}2\theta -{R}_{c}\right\}+{R}_{c}$$

After simplifying, the shear stress at the elastic–plastic boundary is,

83 $${\tau }_{i}=\frac{\xi -1}{4\xi }\left(1+\lambda \right)P\mathit{tan}2\theta +\frac{\xi -1}{2\xi }P\left(1-\lambda \right)\mathrm{sin}2\theta -\frac{\xi -1}{4\xi }{R}_{c}\mathit{tan}2\theta +\frac{{R}_{c}}{2}\frac{\xi +1}{\xi }$$

When $\uplambda =1$, ${\tau }_{r\theta }^{*}={\tau }_{i}=0$, the shear and radial stresses at the elastic–plastic boundary can be expressed as:

84 $${\tau }_{i}=\frac{\xi -1}{4\xi }P\left(\lambda -1\right)\left[\mathit{tan}2\theta -2\mathrm{sin}2\theta \right]$$

85 $${P}_{i}=\frac{1}{\xi +1}\left\{P\left[\left(1+\uplambda \right)+2\left(1-\uplambda \right)\mathit{cos}2\theta \right]-\frac{\xi -1}{2\xi }P\left(\lambda -1\right)\left[1-2\mathit{cos}2\theta \right]-{R}_{c}\right\}$$

Then at the elastic–plastic boundary $r={R}_{*}$, there is ${\sigma }_{r}^{e}+{\sigma }_{\theta }^{e}={\sigma }_{r}^{p}+{\sigma }_{\theta }^{p}$, that is

86 $$ \begin{aligned} & \left[\left(1+\lambda \right)-\left(1-\lambda \right)\mathit{cos}2\theta +\frac{2{P}_{n}}{{R}_{c}}\left(\xi -1\right)\right]{\alpha }^{2-2\upxi }\\ &\quad-\left\{\frac{\xi -1}{\xi +1}\frac{2}{{R}_{c}}\left[P\left(1+\uplambda \right)+2P\left(1-\uplambda \right)\mathit{cos}2\theta -\frac{\xi -1}{2\xi }P\left(\lambda -1\right)+\frac{\xi -1}{\xi }P\left(\lambda -1\right)\mathit{cos}2\theta -{R}_{c}\right]\right.\\&\quad \left.+\left(1+\lambda \right)-4\left(1-\lambda \right)\mathit{cos}2\theta \vphantom{\frac{\xi -1}{2\xi }}\right\}{\alpha }^{1-\upxi }-3\left(1-\lambda \right)\mathit{cos}2\theta =0\end{aligned} $$

If

87 $$A=\left(1+\lambda \right)-\left(1-\lambda \right)\mathit{cos}2\theta +\left(\xi -1\right)\frac{2{P}_{n}}{{R}_{c}}$$

88 $$ \begin{aligned}B & =-\left\{\frac{\xi -1}{\xi +1}\frac{2}{{R}_{c}}\left[P\left(1+\uplambda \right)+2P\left(1-\uplambda \right)\mathit{cos}2\theta -\frac{\xi -1}{2\xi }P\left(\lambda -1\right)\right. \right.\\&\quad \left. \left. +\frac{\xi -1}{\xi }P\left(\lambda -1\right)\mathit{cos}2\theta -{R}_{c}\right]+\left(1+\lambda \right)-4\left(1-\lambda \right)\mathit{cos}2\theta \right\}\end{aligned} $$

89 $$C=-3\left(1-\lambda \right)\mathit{cos}2\theta $$

Then the solution of the original equation can also be expressed in the form of Eqs. (73–75). When $\uplambda =1$, the original Eq. (86) is consistent with the exact solution of Xia et al.^36^ under axisymmetric conditions.

### Stress and displacement of elastic zone

The stress component of the elastic zone can be obtained by putting ${P}_{i}$ and ${\tau }_{i}$ from Eqs. (64–65) or Eqs. (84–85) into Eqs. (58–60). The displacement component of the elastic zone under the non-axisymmetric condition with internal load can be obtained by substituting ${r}_{0}={R}_{*},\alpha =\frac{{R}_{*}}{r},q={P}_{i},\tau ={\tau }_{i}$ into Eqs. (39–40).

90 $${u}_{r}^{e}=\frac{1+\mu }{E}\frac{{R}_{*}^{2}}{r}\left\{\frac{P}{2}\left(\lambda +1\right)+\frac{P}{2}\left[4\left(1-\mu \right)-{\alpha }^{2}\right]\left(\lambda -1\right)\mathit{cos}2\theta -{P}_{i}+2{\tau }_{i}\left[\left(1-\mu \right)-\frac{1}{3}{\alpha }^{2}\right]\mathit{cot}2\theta \right\}$$

91 $${u}_{\theta }^{e}=\frac{1+\mu }{E}\frac{{R}_{*}^{2}}{r}\left\{\frac{P}{2}\left[2\left(2\mu -1\right)-{\alpha }^{2}\right]\left(\lambda -1\right)\mathit{sin}2\theta +\left[\left(2\mu -1\right)-\frac{2}{3}{\alpha }^{2}\right]{\tau }_{i}\right\}$$

When $r={R}_{*}$, the displacement component at the elastic–plastic boundary can be obtained.

92 $${u}_{r}^{e}=\frac{1+\mu }{E}{R}_{*}\left\{\frac{P}{2}\left(\lambda +1\right)+\frac{P}{2}\left(3-4\mu \right)\left(\lambda -1\right)\mathit{cos}2\theta -{P}_{i}+2{\tau }_{i}\left(\frac{2}{3}-\mu \right)\mathit{cot}2\theta \right\}$$

93 $${u}_{\theta }^{e}=\frac{1+\mu }{E}{R}_{*}\left\{\frac{P}{2}\left(4\mu -3\right)\left(\lambda -1\right)\mathit{sin}2\theta +\left(2\mu -\frac{5}{3}\right){\tau }_{i}\right\}$$

When $\uplambda =1$, $r={R}_{*}$, $\alpha =\frac{{R}_{*}}{r},r>{R}_{*}$, the stress and displacement components are as follow,

94 $${P}_{i}=P\left(1-\mathrm{sin}\varphi \right)-C\mathrm{cos}\varphi $$

95 $${\tau }_{i}=0$$

96 $${\mu }_{0}=\frac{2}{P}\left[P\left(1-\mathrm{sin}\varphi \right)-C\mathrm{cos}\varphi \right]$$

97 $${\mu }_{0}^{\mathrm{^{\prime}}}=0$$

98 $${\sigma }_{r}^{e}=P-\left(P\mathrm{sin}\varphi +C\mathrm{cos}\varphi \right){\left(\frac{{R}_{*}}{r}\right)}^{2}$$

99 $${\sigma }_{\theta }^{e}=P+\left(P\mathrm{sin}\varphi +C\mathrm{cos}\varphi \right){\left(\frac{{R}_{*}}{r}\right)}^{2}$$

100 $${\tau }_{r\theta }^{e}=0$$

101 $${u}_{r}^{e}=\frac{1+\mu }{E}\frac{{R}_{*}^{2}}{r}\left\{P\mathrm{sin}\varphi +C\mathrm{cos}\varphi \right\}$$

102 $${u}_{\theta }^{e}=0$$

The above results are consistent with the exact axisymmetric solution^36^. Of course, the plastic zone radius ${R}_{*}$ can also be obtained by Eq. (68) or Eq. (86). Bringing it into the stress component of the plastic zone Eqs. (51–52) and (57) can obtain the radial and shear stress, ${P}_{i}$ and ${\tau }_{i}$, at the elastic–plastic boundary. Then bringing ${P}_{i}$ and ${\tau }_{i}$ into Eqs. (58–60) can obtain the stress component of the elastic zone. And bring ${P}_{i}$ and ${\tau }_{i}$ into Eqs. (90–91) can obtain the displacement component of the elastic zone.

### Displacement of plastic zone

Assuming that under the small deformation condition, the volume of the plastic zone does not change, then there is

103 $$\varepsilon ={\varepsilon }_{1}^{p}+{\varepsilon }_{2}^{p}+{\varepsilon }_{3}^{p}=0$$

Bringing the geometric equation into the above equation can obtain the following equation,

104 $$\left(\frac{\partial {u}_{r}}{\partial r}+\frac{{u}_{r}}{r}\right)+\left(\frac{1}{r}\frac{\partial {u}_{\theta }}{\partial \theta }-\frac{{u}_{\varphi }}{r}\right)+\left(\frac{1}{r}\frac{\partial {u}_{r}}{\partial \theta }+\frac{{\partial u}_{\theta }}{\partial r}\right)=0$$

To make the above equation always hold, it can be separated into the following three equations,

105 $$\frac{\partial {u}_{r}}{\partial r}+\frac{{u}_{r}}{r}=0$$

106 $$\frac{1}{r}\frac{\partial {u}_{\theta }}{\partial \theta }-\frac{{u}_{\theta }}{r}=0$$

107 $$\frac{1}{r}\frac{\partial {u}_{r}}{\partial \theta }+\frac{{\partial u}_{\theta }}{\partial r}=0$$

The solutions of the first and second differential equations are,

108 $${u}_{r}=\frac{A}{r}$$

109 $${u}_{\theta }=B{e}^{\theta }$$

And the solutions also satisfy the third differential equation. That is, the displacement expression of the plastic zone is as above. The deformation coordination condition at the elastic–plastic boundary,

110 $${{u}^{e}=u}^{p}$$

Substituting Eqs. (92–93) into Eq. (110), the constants $A$ and $\mathrm{B}$ can be solved, and then the displacement component of the plastic zone can be obtained as flow,

111 $${u}_{r}=\frac{1+\mu }{E}\frac{{{R}_{*}}^{2}}{r}\left\{\frac{P}{2}\left(\lambda +1\right)+\frac{P}{2}\left(3-4\mu \right)\left(\lambda -1\right)\mathit{cos}2\theta -{P}_{i}+2{\tau }_{i}\left(\frac{2}{3}-\mu \right)\mathit{cot}2\theta \right\}$$

112 $${u}_{\theta }=\frac{1+\mu }{E}{R}_{*}\left\{\left(4\mu -3\right)\frac{P}{2}\left(\lambda -1\right)\mathit{sin}2\theta +\left(2\mu -\frac{5}{3}\right){\tau }_{i}\right\}$$

## Verification and discussion

Modified Fenner equation or Castner equation can only consider the axisymmetric conditions. Yu’ solution^2^ is similar to them, except that the plastic condition form is different. Li’ solution^1^ considers the concept of the construction process and plastic modulus. For the non-axisymmetric classical solution, the plastic zone solution of Yu et al.^2^ is as shown in the follow Eqs. (113–115), and it does not satisfy the plasticity condition, and the intermediate variable ${\sigma }_{2}^{p}$ expression is incorrectly deduced or printed. It cannot be degenerated to the axisymmetric problem, and the circumferential stress may appear anomaly in the plastic zone. And the stress solution of the elastic zone is shown in the following Eqs. (116–118), and the influence of the shear stress at the elastic–plastic boundary on the radial and circumferential stress of the elastic zone is not considered.

113 $${\sigma }_{r}^{p}=\frac{p}{2}\left(1-{\alpha }^{2}\right)\left(1+\lambda \right)-\frac{p}{2}\left(1-4{\alpha }^{2}+{3\alpha }^{4}\right)\left(1-\lambda \right)\mathrm{cos}2\theta $$

114 $${\sigma }_{\theta }^{p}=\frac{\left[\left(\xi +1\right)+\left(\xi -1\right)\mathrm{cos}2\theta \right]{\sigma }_{r}^{p}+2{R}_{c}\mathrm{cos}2\theta }{\left(\xi +1\right)-\left(\xi -1\right)\mathrm{cos}2\theta }$$

115 $${\tau }_{r\theta }^{p}=\frac{\left[\left(\xi -1\right){\sigma }_{r}^{p}+{R}_{c}\right]\mathrm{sin}2\theta }{\left(\xi +1\right)-\left(\xi -1\right)\mathrm{cos}2\theta }$$

116 $${\sigma }_{r}=\frac{1}{2}P\left[\left(1+\uplambda \right)\left(1-{\alpha }^{2}\right)-\left(1-\uplambda \right)\left(1+3{\alpha }^{4}-4{\alpha }^{2}\right)\mathit{cos}2\theta +{\mu }_{0}{\alpha }^{2}\right]$$

117 $${\sigma }_{\theta }=\frac{1}{2}P\left[\left(1+\uplambda \right)\left(1+{\alpha }^{2}\right)+\left(1-\uplambda \right)\left(1+3{\alpha }^{4}\right)\mathit{cos}2\theta -{\mu }_{0}{\alpha }^{2}\right]$$

118 $${\tau }_{r\theta }=\frac{1}{2}P\left[\left(1-\uplambda \right)\left(1+{2\alpha }^{2}-3{\alpha }^{4}\right)\mathrm{sin}2\theta +{\mu }_{0}^{\mathrm{^{\prime}}}{\alpha }^{2}\right]$$

The plastic zone boundary approximate solution of Yu et al.^2^ is shown in the follow Eq. (119).

$${\mathrm{cos}}^{2}2\theta +\frac{2}{\omega }\left[\frac{1+\lambda }{4\left(1-\lambda \right)}\left(1-2{a}^{2}+3{a}^{4}\right)-\frac{\left(1+\lambda +\frac{2X}{p}\right){\mathrm{sin}}^{2}\varphi }{2\left(1-\lambda \right)}\right]\mathrm{cos}2\theta -\frac{1}{\omega }\frac{{\left(1+\lambda \right)}^{2}{a}^{2}}{{4\left(1-\lambda \right)}^{2}}-\frac{1}{\omega }\frac{{\left(1+2{a}^{2}-3{a}^{4}\right)}^{2}}{4{a}^{2}}+\frac{1}{\omega }\frac{\left(1+\lambda +\frac{2X}{p}\right){\mathrm{sin}}^{2}\varphi }{4{a}^{2}{\left(1-\lambda \right)}^{2}}=0$$

119 $$\left(1+\frac{c\mathrm{cot}\varphi }{p}\right){\mathrm{sin}}^{2}\varphi =2{a}^{4}$$

where, $$\omega ={a}^{2}{\mathrm{sin}}^{2}\varphi +2-3{a}^{2}$$, $$X=c\mathrm{cot}\varphi $$, $$a=\frac{{r}_{0}}{{R}_{*}}$$, $$\lambda =\frac{{\sigma }_{x}}{{\sigma }_{z}}$$.

The elastic zone solution of Yu et al.^2^ used the modified approximation calculation of the plastic zone boundary, may result in a smaller plastic zone radius due to ignore the influence of the high-order small quantity. Moreover, none of the above solutions considers the internal load, nor does give a clear solution of the excavation disturbance displacement field. These problems have been perfectly solved in the new solution of this article. The exact solution for the complex non-axisymmetric problems can be obtained by numerical methods. Therefore, the accuracy of the new analytical solution will be verified and discussed through the numerical experiments.

### Model and parameters

**Figure 2:**
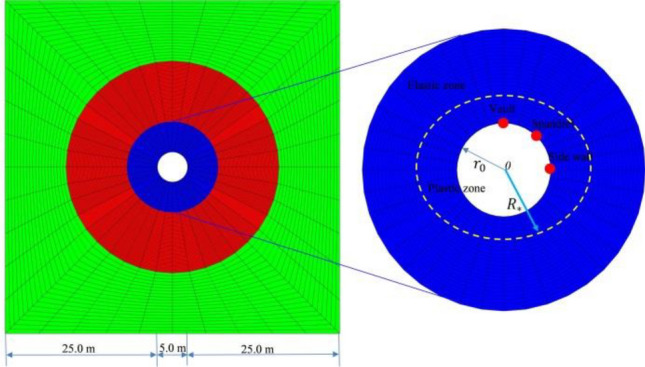
Ideal tunnel model.

FLAC3D software is used and an ideal tunnel model is carried out as shown in Fig. 2. The tunnel radius is 2.5 m, and the buried depth is 300 m. The influence range of the tunnel excavation is considered to be 5 times the diameter of the tunnel. The lateral boundary conditions were normally constrained. The in-situ stress assigned to the model is primarily composed of gravitational stress and characterized by a lateral pressure coefficient. And the underground tunnels in the model were simulated by full-face excavation. The surrounding rock is simulated by solid elements. The simulation employs the M-C constitutive model. The specific parameters of the surrounding rock are shown in Table 1.

**Table 1:** Test parameters.

Materials	Volume weight (kN/m^3^)	Deformation modulus (GPa)	Poisson's ratio	Bulk modulus (GPa)	Shear modulus (GPa)	Cohesion (MPa)	Friction angle (°)
Rock mass	25	6.0	0.3	5.0	2.3	0.7	39

Based on the above research ideas and corresponding analytical equations, programming can directly obtain the theoretical distribution characteristics on the stress field, the displacement field and the plastic zone radius. The new solution of this paper, the numerical experiment and the analytical solution Eqs. (113–115) of Yu et al.^2^ for the circular tunnel under non-axisymmetrical conditions with internal load are compared and verified on the plastic zone, stress field, displacement field, etc. And methods 1, 2, 3 and 4 are the numerical solution, the modified Castner solution, the new solution 1 and the new solution 2 in this article, respectively. And the influence of the lateral pressure coefficient $\lambda$ (0.5, 1.0, 1.5) and internal load ${P}_{n}$ (0.0, 0.1 MPa) in the result are mainly considered.

### Plastic zone characteristics

**Figure 3:**
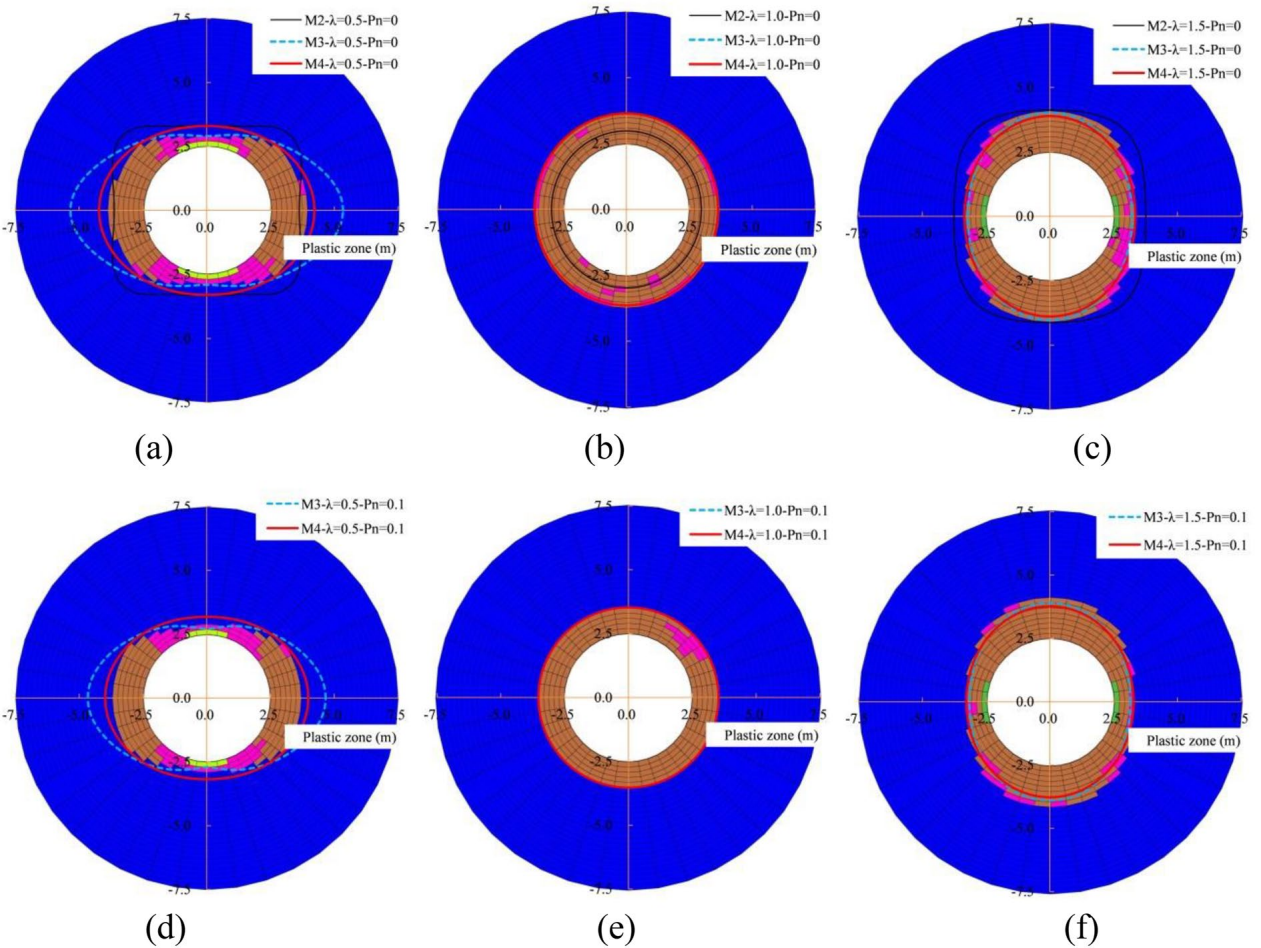
Distribution characteristics of the plastic zone, (**a**) $\lambda =0.5$, (**b**) $\lambda =1.0$ and (**c**) $\lambda =1.5$ when ${P}_{n}=0.0 MPa$; (**d**)$\lambda =0.5$, (**e**)$\lambda =1.0$ and (**f**) $\lambda =1.5$ when ${P}_{n}=0.1 \mathrm{MPa}$.

The distribution characteristics and values of the plastic zone under different lateral pressure coefficients are shown in Fig. 3 and Table 2. The shape of the plastic zone shows an ellipse-circle-ellipse change trend as the lateral pressure coefficient increases. The differences on the plastic zone of the four methods are relatively more obvious, when the lateral pressure coefficient is less than 1.0. Methods 3 and 4 both show a flat ellipse shape and are similar to the numerical test, while method 2 has a four stars shape trend. If the lateral pressure coefficient is equal to 1.0, the plastic zone all presents a circular shape consistent with the distribution of the classical solution of Xia et al.^15^, only there is the values difference. Methods 3 and 4 both show a vertical ellipse shape and are in good agreement with the numerical test, while method 2 has a four stars shape trend, when the lateral pressure coefficient is more than 1.0.

**Table 2:** Comparative analysis of plastic zone radius under different methods (m).

Internal load (MPa)	Lateral pressure coefficient	0.5				1.0				1.5			
	Methods	1	2	3	4	1	2	3	4	1	2	3	4
0.0	Vault	2.90	3.33	2.90	3.30	3.70	2.98	3.64	3.64	4.10	4.12	4.02	3.89
	Spandrel	3.70	4.32	3.76	3.70	3.70	2.98	3.64	3.64	3.70	4.40	3.59	3.60
	Side wall	3.90	3.62	5.35	4.22	3.70	2.98	3.64	3.64	3.30	3.76	3.22	3.35
	Maximum error (%)	–	17	37	14	–	19	2	2	–	19	3	5
0.1	Vault	2.90		2.71	3.18	3.50		3.53	3.53	4.10		3.90	3.77
	Spandrel	3.50		3.57	3.55	3.50		3.53	3.53	3.70		3.51	3.51
	Side wall	3.70		4.66	3.98	3.50		3.53	3.53	3.30		3.16	3.28
	Maximum error (%)	–		26	10	–		1	1	–		5	8

Under the condition of no internal load and the lateral pressure coefficient is 1.0, the plastic zone radius (3.64 m) of the new method is very close to the plastic zone radius (3.70 m) of the numerical test, and the maximum error does not exceed 2%. While the plastic zone radius of the modified Castner solution is 2.90 m, and the maximum error is 19%. When the lateral pressure coefficient is 0.5, the plastic zone radius of the vault, Spandrel, and side wall are 3.30 m, 3.70 m and 4.22 m, respectively, for the new method 4. And they are 2.90 m, 3.70 m and 3.30 m, respectively, for the numerical test. Their maximum error does not exceed 14%, while the errors of methods 2 and 3 are relatively larger. When the lateral pressure coefficient is 1.5, the plastic zone radius of the vault, Spandrel, and side wall are 3.89 m, 3.60 m, and 3.35 m, respectively, for the new method 4. And they are 4.10 m, 3.70 m, 3.90 m, respectively, for the numerical test. Their maximum error does not exceed 5%, while the error of method 2 is 19%. The distribution law of the plastic zone considering the internal load of 0.1 MPa is consistent with the shape without internal load, only the value is smaller. The maximum errors of method 4 are 10%, 1%, and 8%, respectively, when the lateral pressure coefficient are 0.5, 1.0 and 1.5. That is, the distribution characteristics and values ​​of the plastic zone obtained by the new method 4 are closer to the numerical test results, it is relatively more accurate.

### Stress characteristics

**Figure 4:**
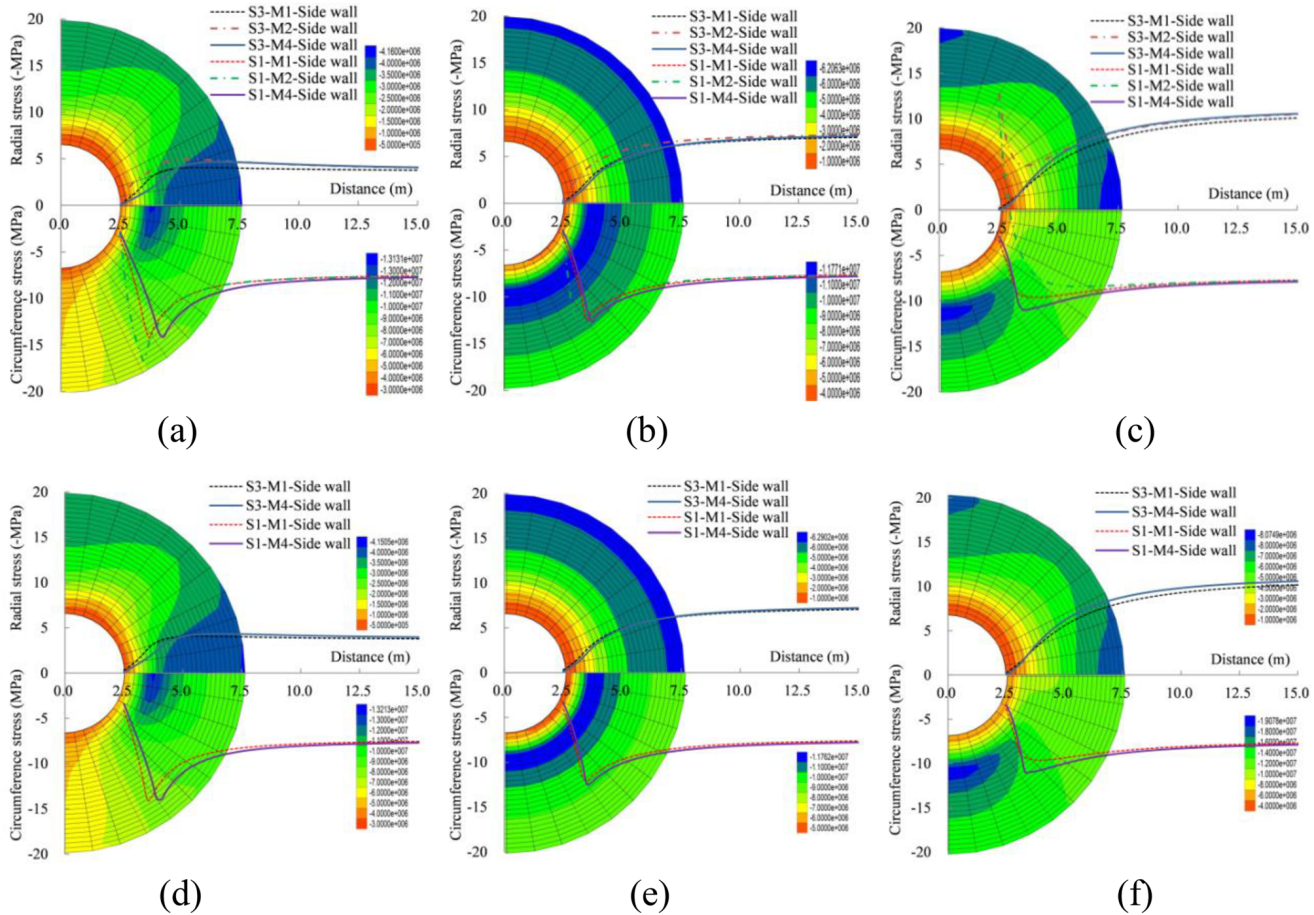
Distribution characteristics of radial and circumferential stress, (**a**)$\lambda =0.5$, (**b**) $\lambda =1.0$ and (**c**)$\lambda =1.5$ when ${P}_{n}=0.0 MPa$; (**d**) $\lambda =0.5$, (**e**)$\lambda =1.0$ and (**f**) $\lambda =1.5$ when ${P}_{n}=0.1 \mathrm{MPa}$.

The analytical solution and numerical test results are compared in terms of the stress characteristics at the side wall, as shown in Fig. 4 and Table 3. The circumference stress is the largest at the plastic zone radius, while the radial stress increases consistently and eventually tends to the initial stress as the distance increases. Meanwhile, the change law of radial stress is also mutually verified with the initial plastic zone radial stress hypothesis of Yu et al.^2^. The crescent-shaped circumference compressive stress nuclei will be formed on the left and right side walls, if the lateral pressure coefficient is 0.5.While the nuclei will be formed on the vault and the bottom, if the lateral pressure coefficient is 1.5. And an annular stress nucleus will be formed when the lateral pressure coefficient is 1.0. A four stars shaped radial stress field distribution will be formed when the lateral pressure coefficient is 0.5 and 1.5, while a ring shaped stress distribution will be formed when the lateral pressure coefficient is 1.0.

**Table 3:** Comparative analysis of stress at the elastic–plastic boundary under different methods (MPa).

Internal load (MPa)	Lateral pressure coefficient	0.5		1		1.5	
	Methods	1	4	1	4	1	4
0.0	Radial stress	3.24	2.90	2.80	2.40	1.67	1.64
	Circumference stress	13.17	14.14	11.85	12.60	9.21	10.13
0.1	Radial stress	3.13	2.96	2.58	2.24	1.95	1.70
	Circumference stress	13.61	13.92	12.08	12.40	9.31	10.21

The stress distribution law of surrounding rock with the internal load is basically the same as that without internal load. Due to the effect of internal load, the plastic zone radius decreases, the circumference stress at the elastic–plastic boundary increases, while the radial stress reduces. The analytical solution of the circumference stress is larger than the numerical test, but the analytical solution of radial stress at the elastic–plastic boundary is smaller than the numerical solution. When the lateral pressure coefficients are 0.5, 1.0 and 1.5 without the internal load, the radial stress are 3.24/2.90, 2.80/2.40 and 1.67/1.64 MPa, respectively, and the maximum error is 14%, for the numerical solution/analytical solution at the elastic–plastic boundary. And the circumference stress are 13.17/14.14, 11.85/12.60 and 9.21/10.13 MPa for them, respectively, and the maximum error is 9%. However, Method 2 has a relatively large error, especially when the lateral pressure coefficient is larger than 1.0. When the lateral pressure coefficients are 0.5, 1.0 and 1.5 with the internal load, the radial stress are 3.13/2.96, 2.58/2.24 and 1.95/1.70 MPa, respectively, and the maximum error is 13%, for the numerical solution/analytical solution at the elastic–plastic boundary. And the circumference stress are 13.61/13.92, 12.08/12.40 and 9.31/10.21 MPa for them, respectively, and the maximum error is 9%. That is, the stress field distribution characteristics and values ​​obtained by the new method 4 in this paper are closer to the numerical test results, it is relatively more accurate.

### Displacement characteristics

**Figure 5:**
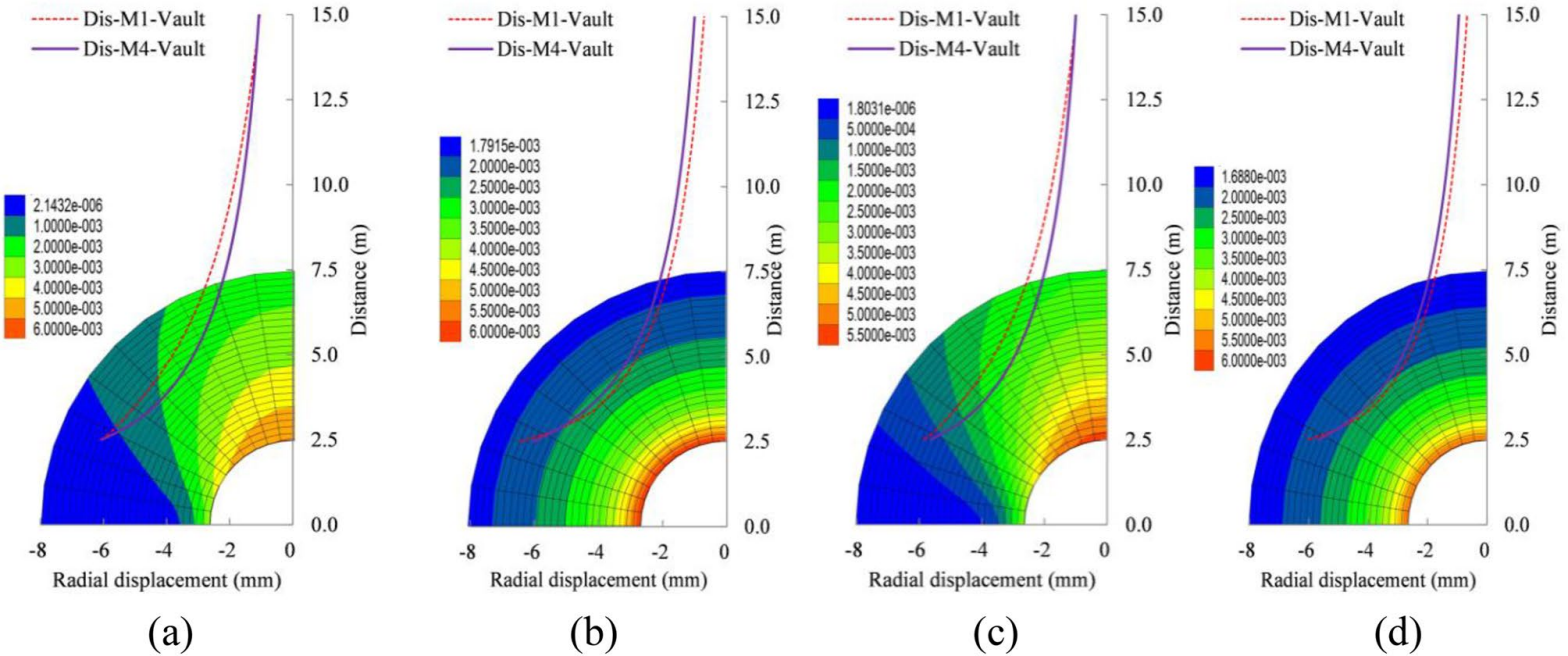
Distribution characteristics of radial displacement, (**a**) $\lambda =0.5$ and (**b**) $\lambda =1.0$ when ${P}_{n}=0.0 \mathrm{MPa}$; (**c**) $\lambda =0.5$ and (**d**)$\lambda =1.0$ when ${P}_{n}=0.1 \mathrm{MPa}$.

The analytical solution and numerical test results are compared in terms of the vault displacement, as shown in Fig. 5 and Table 4. All the radial displacement curves exhibit the same variation trend on analytical solutions and numerical experiments. The radial displacement of surrounding rock decreases with the increase of distance. When the lateral pressure coefficients are 0.5 and 1.0 without the internal load, the radial displacement of vault are 6.10/6.06 and 6.46/6.05 mm, respectively, for the numerical solution/analytical solution. And the local maximum error is no more than 6%. When the lateral pressure coefficients are 0.5 and 1.0 with the internal load, the radial displacement of vault are 5.84/5.63 and 6.01/5.67 mm, respectively, for the numerical solution/analytical solution. And the local maximum error is no more than 6%. The comparisons confirm that the analytical solution deformation agree well with the numerical test results in the space and values.

**Table 4:** Comparison on the radial displacement of vault (mm).

Methods		Lateral pressure coefficient					
		0.5			1		
		1	4	Maximum error (%)	1	4	Maximum error (%)
Internal load (MPa)	0.0	6.10	6.06	1	6.46	6.05	6
	0.1	5.84	5.63	4	6.01	5.67	6

Overall, both the distribution characteristics and magnitudes of displacement, stress and plastic zone obtained by analytic solution are in good agreement with numerical test. There is only a slight different in value, due to the plastic zone solution is obtained on the basis of certain assumptions. It proves that the new analytic solution method 4 is reliable and accurate.

### Discussion

For the theoretical analysis, the tunnel surrounding rock material can be regarded as a continuous, uniform and isotropic ideal elastoplastic medium, the tunnel is a deep-buried cavern, and the initial stress only considers the influence of the self-weight stress. However, the surrounding rock and tunnel conditions in the actual project are complex and changeable. And different numerical analysis tools have different results. Therefore, this section carries out similar analyzes on the different buried depths, $\mathrm{H}$ (30 m, 100 m, 200 m, 300 m), different deformation modulus, $\mathrm{E}$ (250 MPa, 500 Mpa, 1000 MPa, 6000 MPa), different lateral pressure coefficient, $\lambda$ (1.5, 2.0, 2.5, 3.0) and the different software program (M1, finite difference method of FLAC3D and M5, finite element method of FINAL). The corresponding results on high ground stress, weak surrounding rock and shallow tunnels based on the original basic scheme parameters are as follows.

**Figure 6:**
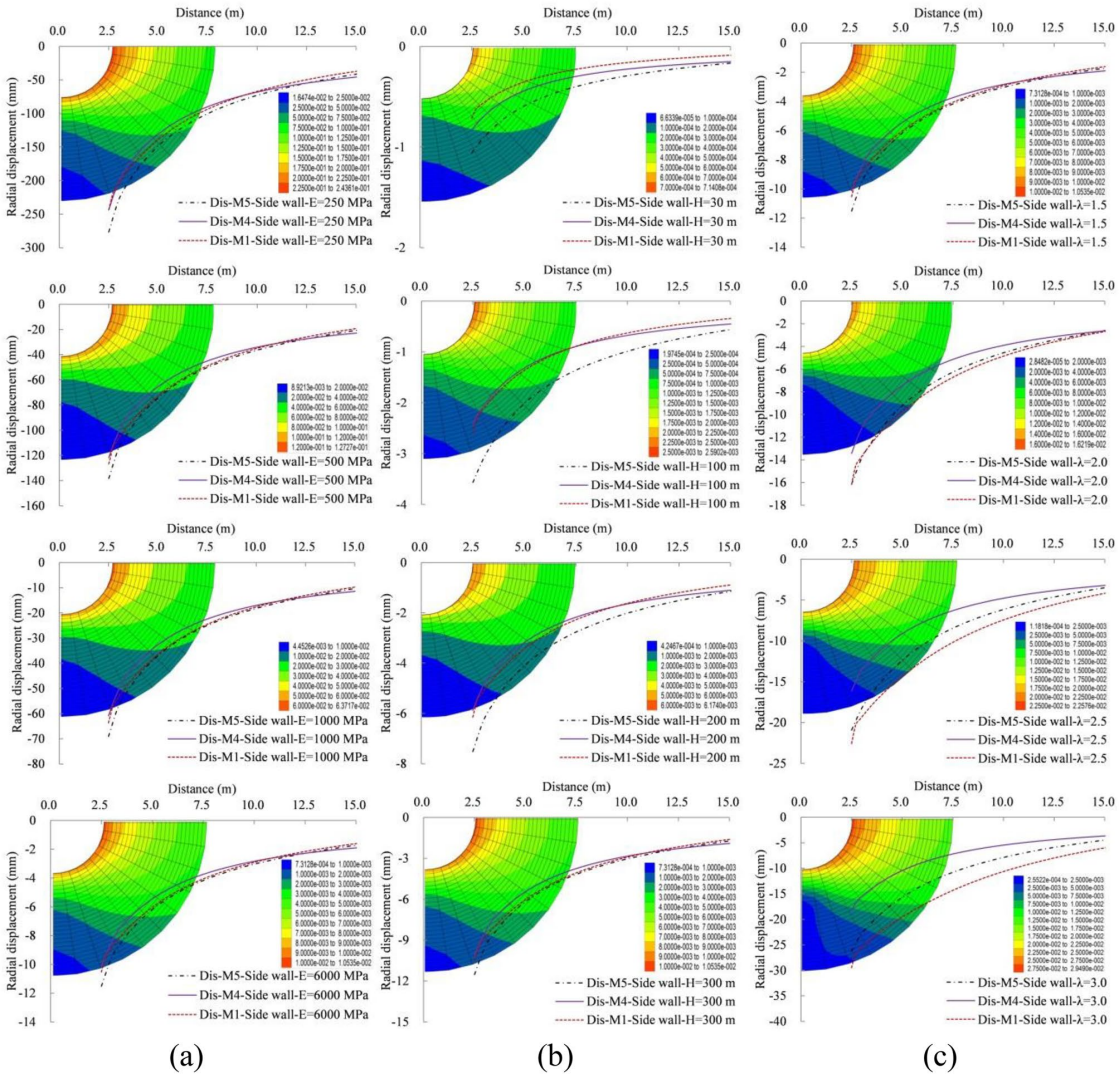
Radial displacement distribution characteristics of the side wall under (**a**) the different deformation modulus, (**b**) the different buried depths and (**c**) the different lateral pressure coefficient.

The displacement distribution characteristics of the side wall under the different deformation modulus, the buried depths and the lateral pressure coefficient are shown in Fig. 6 and Table 5. The side wall displacement distribution laws of the three methods are in good agreement, and there is only difference in the value. For different deformation modulus, the larger buried depth and the smaller lateral pressure coefficient, there are the trend of method 5 > method 1 > method 4. And the maximum error of the analytical solution method and the finite difference method is no more than 5%, 7% and 4%, respectively. However, it shows the trend of method 5 > method 4 > method 1 for a deeper buried depth tunnel. It shows the trend of method 1 > method 5 > method 4 for a larger lateral pressure coefficient. And the larger the lateral pressure coefficient is, the larger the relative error of the analytical solution is. The source of the analytical solution error is related to the basic assumptions. That is to say, the accuracy of the displacement analytical solution is higher for a deep buried approximate axisymmetric tunnel.

**Table 5:** Comparison on the radial displacement of side wall and the circumferential stress of vault.

Methods		Location					
		Radial displacement of side wall (mm)			Circumferential stress of vault (MPa)		
		M1	M4	M5	M1	M4	M5
Deformation modulus (GPa)	250	243.57	243.50	276.50	19.88	18.55	17.48
	500	126.80	121.75	138.30	19.88	18.55	17.48
	1000	63.70	60.88	69.08	19.88	18.55	17.48
	6000	10.53	10.15	11.53	19.88	18.55	17.48
Buried depths (m)	30	0.71	0.83	1.06	2.39	2.33	2.05
	100	2.59	2.42	3.56	6.99	6.55	5.85
	200	6.15	5.88	7.52	13.38	12.99	11.76
	300	10.53	10.15	11.53	19.88	18.55	17.48
Lateral pressure coefficient	1.5	10.53	10.15	11.53	19.88	18.55	17.48
	2.0	16.17	13.44	16.14	26.94	24.15	22.96
	2.5	22.58	16.15	20.92	32.88	30.29	27.87
	3.0	29.49	18.23	26.24	38.57	36.94	34.04

**Figure 7:**
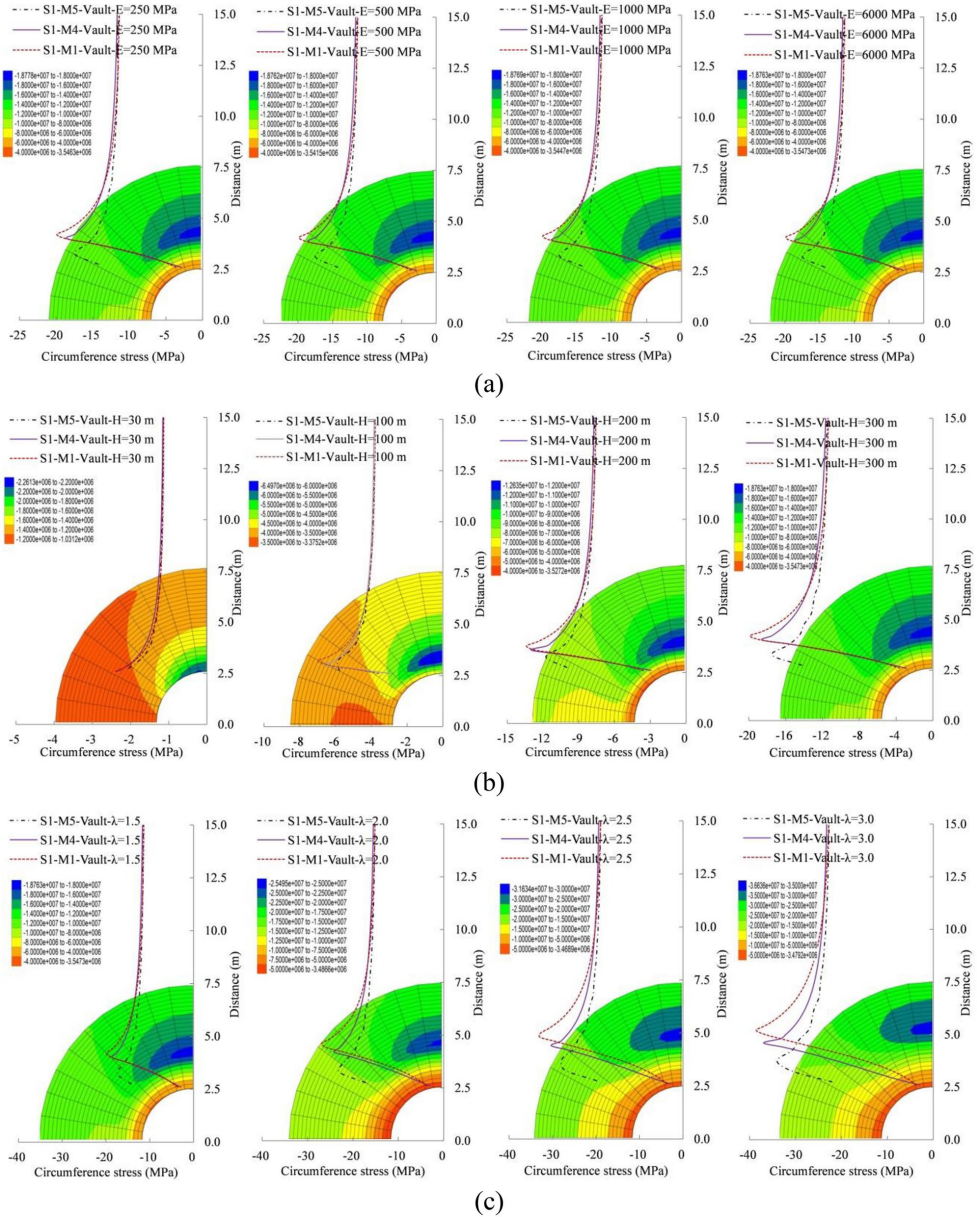
Circumferential stress distribution characteristics of the vault under (**a**) the different deformation modulus, (**b**) the different buried depths and (**c**) the different lateral pressure coefficient.

The stress distribution characteristics of the vault under the different deformation modulus, the buried depths and the lateral pressure coefficient are shown in Fig. 7 and Table 5. The vault circumferential stress distribution characteristics of the three methods are in good agreement, and there is only difference in the value. And the maximum circumferential stress of the vault stress core shows the trend of method 5 > method 4 > method 1. The vault circumferential stresses are equal under different deformation modulus, which means that the deformation parameters do not affect the stress distribution. And it is consistent with the basic inference in elastic mechanics. The maximum errors of the analytical solution method and the finite difference method are not more than 7% for different buried depths. And the greater the buried depth is, the smaller the relative error of the circumferential stress is. The maximum error of the analytical solution method and the finite element method is not more than 8% for different lateral pressure coefficients. And the larger the lateral pressure coefficient is, the greater the relative error of the circumferential stress is. In other words, the stress analytical solution is more accurate for the deep-buried near-axisymmetric conditions.

**Figure 8:**
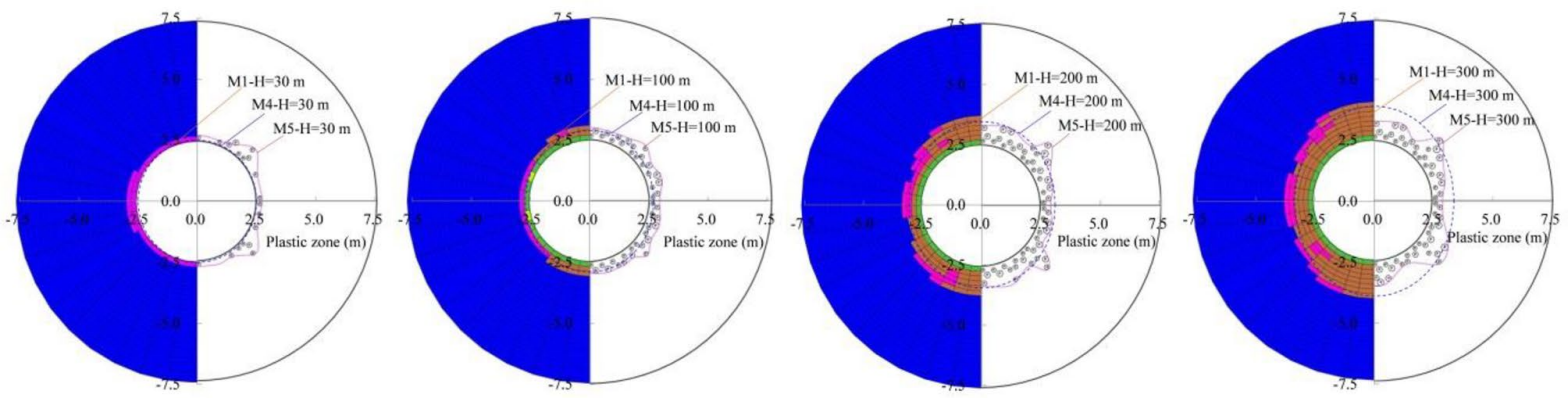
Plastic zone distribution characteristics under the different buried depths.

The plastic zone distribution characteristics under the different buried depths are shown in Fig. 8 and Table 6. The distribution laws of the plastic zone of the three methods are basically the same. The difference of the plastic zone radius around the tunnel is inevitable due to the differences in element division methods, excavation simulation methods, calculation methods, etc. The larger the buried depth is, the larger the plastic zone radius is, the smaller the plastic zone radius error at the key part is. And the maximum error of the analytical solution method and the finite difference method is not more than 8%. The deformation parameters only affect the distribution of the deformation field and do not influence the plastic zone distribution. The greater the lateral pressure coefficient is, the greater the relative error of the plastic zone radius is. If the lateral pressure coefficient is less than 2.0, and the maximum error of the plastic zone radius for the analytical solution method and the finite difference method of the vault is not more than 10%. The plastic zone of the side wall on the finite difference method has obvious display error when the lateral pressure coefficient is larger. The research results of Kabwe et al. (2020) show that when the lateral pressure coefficient is greater than 2.0, a butterfly-shaped plastic zone will appear on the spandrel. This phenomenon is correct. In this paper, a simplified plastic yield condition is adopted, which mainly considers the plastic zone of side walls and vaults, so the butterfly-shaped plastic zone is not obvious.

**Table 6:** Comparison on the plastic zone radius (m).

Methods		Location					
		Vault			Side wall		
		M1	M4	M5	M1	M4	M5
Deformation modulus (GPa)	250	4.10	3.89	3.32	3.70	3.35	2.94
	500	4.10	3.89	3.32	3.70	3.35	2.94
	1000	4.10	3.89	3.32	3.70	3.35	2.94
	6000	4.10	3.89	3.32	3.70	3.35	2.94
Buried depths (m)	30	2.70	2.50	2.73	2.90	2.50	2.75
	100	3.10	2.89	2.98	2.90	2.70	2.93
	200	3.70	3.46	3.31	3.30	3.09	2.94
	300	4.10	3.89	3.32	3.70	3.35	2.94
Lateral pressure coefficient	1.5	4.10	3.89	3.32	3.70	3.35	2.94
	2.0	4.50	4.09	3.71	4.10	3.16	2.92
	2.5	4.90	4.27	3.91	5.50	3.00	2.93
	3.0	5.30	4.42	3.91	7.10	2.85	2.89

The analytical solution in this paper is obtained on the basis of assumptions, so for the weak surrounding rock, the high ground stress and the shallow tunnels, there must be errors in the solution. However, the accuracy is still relatively higher for a deep-buried approximate axisymmetric tunnel.

## Conclusions

Analytical solution is a powerful mean to analyze tunnel engineering problems, however the existing solutions fail to directly obtain the secondary stress field or excavation disturbance displacement field that we care about. The existing non-axisymmetric elastic–plastic solutions are mostly implicit and the approximate solution and cannot be degenerated to an accurate axisymmetric expression. So we derive the elastic–plastic analytical solution of a circular tunnel under non-axisymmetric external loads with radial inner load. The main conclusions are as follows (Supplementary Information S1):

1. The elastic solution of circular tunnel under the condition of non-axisymmetric external load with the radial and shear inner loads is deduced.
2. The new elastic–plastic solution and the plastic zone radius equation of the circular tunnel under the condition of non-axisymmetric external load with radial inner load are derived.
3. The new method can directly obtain the secondary stress field and excavation disturbance displacement field, and can degenerate to an accurate axisymmetric expression.

Both the distribution characteristics and values of displacement, stress and plastic zone obtained by the new analytic solution are in good agreement with numerical test in space, indicating that the new solution can provide a relatively accurate theoretical basis for the analysis and research of tunnel engineering.

## Supplementary Information

Supplementary Information.
