# Supplementary material for: An new elastic–plastic analytical solution of circular tunnel under non-axisymmetric conditions
Source: Sci Rep. 2022 Mar 14;12:4367. doi: 10.1038/s41598-022-08353-3 (PMC8921244; doi:10.1038/s41598-022-08353-3)
Supplement: Supplementary file 1 — Supplementary Information. [file 41598_2022_8353_MOESM1_ESM.docx]

# Appendix A. Stress compatibility equation

For the stress solution method of the plane elastic problem, the stress component is the basic unknown function, and its core is to solve the stress function or the biharmonic equation. The basic equations in the cartesian coordinate system are as follows.

Balance equations,

$\frac{\partial\sigma_{x}}{\partial_{x}}+\frac{\partial\tau_{yx}}{\partial_{y}}+f_{x}=0$  (A122)

$\frac{\partial\sigma_{y}}{\partial_{y}}+\frac{\partial\tau_{xy}}{\partial_{x}}+f_{y}=0$ (A123)

Geometric equations,

$\varepsilon_{x}=\frac{\partial u}{\partial x}$ (A124)

$\varepsilon_{y}=\frac{\partial v}{\partial y}$ (A125)

$\gamma_{xy}=\frac{\partial v}{\partial x}+\frac{\partial u}{\partial y}$ (A126)

Physical equations,

$\varepsilon_{x}=\frac{1}{E}\left( \sigma_{x}-\mu\sigma_{y} \right)$ (A127)

$\varepsilon_{y}=\frac{1}{E}\left( \sigma_{y}-\mu\sigma_{x} \right)$ (A128)

$\gamma_{xy}=\frac{2\left( 1+\mu\right)}{E}\tau_{xy}$ (A129)

The displacement component is eliminated from the geometric Eqs. (A124~A126), that is, each strain component is differentiated with respect to x, y, and xy, then,

$\frac{{\partial^{2}\varepsilon}_{x}}{\partial y^{2}}=\frac{\partial^{3}u}{\partial x\partial y^{2}}$ (A130)

$\frac{{\partial^{2}\varepsilon}_{y}}{\partial x^{2}}=\frac{\partial^{3}v}{\partial y\partial x^{2}}$ (A131)

$\frac{{\partial^{2}\gamma}_{xy}}{\partial y\partial x}=\frac{\partial^{3}v}{\partial x^{2}\partial y}+\frac{\partial^{3}u}{\partial y^{2}\partial x}$ (A132)

Adding the above Eqs. (A130~A131) can get the deformation coordination equation,

$\frac{{\partial^{2}\varepsilon}_{x}}{\partial y^{2}}+\frac{{\partial^{2}\varepsilon}_{y}}{\partial x^{2}}=\frac{\partial^{3}u}{\partial x\partial y^{2}}+\frac{\partial^{3}v}{\partial y\partial x^{2}}=\frac{{\partial^{2}\gamma}_{xy}}{\partial y\partial x}$ (A133)

Bring the physical Eqs. (A127~A129) into the deformation coordination equation can obtain the coordination equation expressed by stress,

$\frac{\partial^{2}}{\partial y^{2}}\left( \sigma_{x}-\mu\sigma_{y} \right)+\frac{\partial^{2}}{\partial x^{2}}\left( \sigma_{y}-\mu\sigma_{x} \right)=2\left( 1+\mu\right)\frac{\partial^{2}}{\partial y\partial x}\tau_{xy}$ (A134)

Deforming the balance Eqs. (A122~A123) and deriving x and y respectively can obtain the following equations,

$\frac{\partial^{2}\tau_{yx}}{\partial_{y}\partial_{x}}=\frac{\partial\left( -\frac{\partial\sigma_{x}}{\partial_{x}}-f_{x} \right)}{\partial_{x}}=-\frac{\partial^{2}\sigma_{x}}{\partial_{x^{2}}}-\frac{\partial f_{x}}{\partial_{x}}$ (A135)

$\frac{\partial^{2}\tau_{xy}}{\partial_{x}\partial_{y}}=\frac{\partial\left( -\frac{\partial\sigma_{y}}{\partial_{y}}-f_{y} \right)}{\partial_{y}}=-\frac{\partial^{2}\sigma_{y}}{\partial_{y^{2}}}-\frac{\partial f_{y}}{\partial_{y}}$ (A136)

Adding the above two equations can get the following equation,

$2\frac{\partial^{2}\tau_{yx}}{\partial_{y}\partial_{x}}=-\frac{\partial^{2}\sigma_{x}}{\partial_{x^{2}}}-\frac{\partial f_{x}}{\partial_{x}}-\frac{\partial^{2}\sigma_{y}}{\partial_{y^{2}}}-\frac{\partial f_{y}}{\partial_{y}}$ (A137)

Putting the above Eq. (A137) into the right side of the coordination Eq. (A134) can get the stress coordination equation,

$\frac{\partial^{2}}{\partial y^{2}}\left( \sigma_{x}-\mu\sigma_{y} \right)+\frac{\partial^{2}}{\partial x^{2}}\left( \sigma_{y}-\mu\sigma_{x} \right)=\left( 1+\mu\right)\left( -\frac{\partial^{2}\sigma_{x}}{\partial_{x^{2}}}-\frac{\partial f_{x}}{\partial_{x}}-\frac{\partial^{2}\sigma_{y}}{\partial_{y^{2}}}-\frac{\partial f_{y}}{\partial_{y}} \right)$ (A138)

Transforming the above Eq. (A138) can get the following equation,

$\frac{\partial^{2}}{\partial y^{2}}\left( \sigma_{x}-\mu\sigma_{y} \right)+\left( 1+\mu\right)\frac{\partial^{2}\sigma_{y}}{\partial_{y^{2}}}+\frac{\partial^{2}}{\partial x^{2}}\left( \sigma_{y}-\mu\sigma_{x} \right)+\left( 1+\mu\right)\frac{\partial^{2}\sigma_{x}}{\partial_{x^{2}}}=-\left( 1+\mu\right)\left( \frac{\partial f_{x}}{\partial_{x}}+\frac{\partial f_{y}}{\partial_{y}} \right)$ (A139)

The above Eq. (A139) can be further simplified to the stress coordination equation or governing equation,

$\left( \frac{\partial^{2}}{\partial y^{2}}+\frac{\partial^{2}}{\partial x^{2}} \right)\left( \sigma_{x}+\sigma_{y} \right)=-\left( 1+\mu\right)\left( \frac{\partial f_{x}}{\partial_{x}}+\frac{\partial f_{y}}{\partial_{y}} \right)$ (A140)

In the case of constant volume force, the stress coordination Eq. (A140) can be simplified to the following expression.

$\left( \frac{\partial^{2}}{\partial y^{2}}+\frac{\partial^{2}}{\partial x^{2}} \right)\left( \sigma_{x}+\sigma_{y} \right)=0=\nabla^{2}\left( \sigma_{x}+\sigma_{y} \right)$ (A141)

The balanced differential equation is a system of non-homogeneous equations, whose solutions are homogeneous general solutions and non-homogeneous special solutions. The special solution can take the following expressions.

$\sigma_{x}=-f_{x}x$ (A142)

$\sigma_{y}=-f_{y}y$ (A143)

$\tau_{xy}=0$ (A144)

According to the compatibility of the partial derivatives, for $f=f\left( x,y \right)$, there is

$\frac{\partial}{\partial x}\left( \frac{\partial f}{\partial y} \right)=\frac{\partial}{\partial y}\left( \frac{\partial f}{\partial x} \right)$ (A145)

If C and D satisfy $\frac{\partial}{\partial x}C=\frac{\partial}{\partial y}D$, then there must be a function $f$, and $\frac{\partial f}{\partial y}=C$，$\frac{\partial f}{\partial x}=D$.

Then the homogeneous balance equation can be rewritten as the following expression.

$\frac{\partial}{\partial_{x}}\left( \sigma_{x} \right)=\frac{\partial}{\partial_{y}}\left( {-\tau}_{yx} \right)$ (A146)

Then, there must be a function, $A\left( x,y \right)$, and $\frac{\partial A}{\partial y}=\sigma_{x}$，$\frac{\partial A}{\partial x}={-\tau}_{yx}$.

Similarly, there is a function, $B\left( x,y \right)$, and $\frac{\partial B}{\partial x}=\sigma_{y}$，$\frac{\partial B}{\partial y}={-\tau}_{xy}$.

According to the theorem of reciprocal shear stress, there is, $\frac{\partial}{\partial x}A=\frac{\partial}{\partial y}B$.

Similarly, according to the compatibility of partial derivatives, there must be a function $\psi\left( x,y \right)$, and $\frac{\partial\psi}{\partial y}=A$，$\frac{\partial\psi}{\partial x}=B$. Then the following equations are given.

$\sigma_{x}=\frac{\partial A}{\partial y}=\frac{\partial}{\partial y}\frac{\partial\psi}{\partial y}=\frac{\partial^{2}\psi}{\partial y^{2}}$ (A147)

$\sigma_{y}=\frac{\partial B}{\partial x}=\frac{\partial}{\partial x}\frac{\partial\psi}{\partial x}=\frac{\partial^{2}\psi}{\partial x^{2}}$ (A148)

$\tau_{yx}=-\frac{\partial A}{\partial x}=-\frac{\partial}{\partial x}\frac{\partial\psi}{\partial y}=-\frac{\partial^{2}\psi}{\partial x\partial y}$ (A149)

Then, the solutions of the inhomogeneous equation are the following equations.

$\sigma_{x}=\frac{\partial^{2}\psi}{\partial y^{2}}-f_{x}x$ (A150)

$\sigma_{y}=\frac{\partial^{2}\psi}{\partial x^{2}}-f_{y}y$ (A151)

$\tau_{yx}=-\frac{\partial^{2}\psi}{\partial x\partial y}$ (A152)

Among them, $\psi\left( x,y \right)$ is called the stress function of the plane problem, also known as the Airy stress function. Only $\psi\left( x,y \right)$ is required to obtain the stress component. The stress function of the above equation is derived from the balanced differential equation, so it should also satisfy the stress compatibility Eq. (A141), then there is

$\left( \frac{\partial^{2}}{\partial y^{2}}+\frac{\partial^{2}}{\partial x^{2}} \right)\left( \frac{\partial^{2}\psi}{\partial y^{2}}-f_{x}x+\frac{\partial^{2}\psi}{\partial x^{2}}-f_{y}y \right)=0$ (A153)

$f_{x}$ and $f_{y}$ are constants, and the derivative is 0, so it can be further simplified as,

$\left( \frac{\partial^{2}}{\partial y^{2}}+\frac{\partial^{2}}{\partial x^{2}} \right)\left( \frac{\partial^{2}\psi}{\partial y^{2}}+\frac{\partial^{2}\psi}{\partial x^{2}} \right)=\left( \frac{\partial^{2}}{\partial y^{2}}+\frac{\partial^{2}}{\partial x^{2}} \right)^{2}\psi=0$ (A154)

The above Eq. (A154) is the stress compatibility equation expressed by the stress function. For plane problem of the constant volume force, the stress method finally comes down to solving a stress function $\psi\left( x,y \right)$ problem.

There are following relationships between the polar coordinate system and the rectangular coordinate system.

Coordinate transformation are, $r^{2}=x^{2}+y^{2}$， $\theta=arctan\frac{y}{x}$ , $x=r\cos\theta$，$y=r\sin\theta$.

Derivative conversion are, $\frac{\partial r}{\partial x}=\frac{2x}{2\sqrt{x^{2}+y^{2}}}=\frac{x}{r}=\cos\theta$，$\frac{\partial r}{\partial y}=\frac{2y}{2\sqrt{x^{2}+y^{2}}}=\frac{y}{r}=\sin\theta$，$\frac{\partial\theta}{\partial x}=\frac{-\frac{y}{x^{2}}}{1+\left( \frac{y}{x} \right)^{2}}=-\frac{y}{r^{2}}=-\frac{\sin\theta}{r}$，$\frac{\partial\theta}{\partial y}=\frac{\frac{1}{x}}{1+\left( \frac{y}{x} \right)^{2}}=\frac{x}{r^{2}}=\frac{\cos\theta}{\rho}$.

Stress function conversion are,

$\frac{\partial\psi}{\partial x}=\frac{\partial\psi}{\partial r}\frac{\partial r}{\partial x}+\frac{\partial\psi}{\partial\theta}\frac{\partial\theta}{\partial x}=\cos\theta\frac{\partial\psi}{\partial r}-\frac{\sin\theta}{r}\frac{\partial\psi}{\partial\theta}$, $\frac{\partial\psi}{\partial y}=\frac{\partial\psi}{\partial r}\frac{\partial r}{\partial y}+\frac{\partial\psi}{\partial\theta}\frac{\partial\theta}{\partial y}=\sin\theta\frac{\partial\psi}{\partial r}+\frac{\cos\theta}{r}\frac{\partial\psi}{\partial\theta}$,

$\frac{\partial^{2}\psi}{\partial x^{2}}=\frac{\partial}{\partial x}\left( \frac{\partial\psi}{\partial x} \right)=\cos^{2} \theta\frac{\partial^{2}\psi}{\partial r^{2}}+\sin^{2} \theta\left( \frac{1}{r}\frac{\partial\psi}{\partial r}+\frac{1}{r^{2}}\frac{\partial^{2}\psi}{\partial\theta^{2}} \right)-2\sin\theta\cos\theta\left[ \frac{\partial}{\partial r}\left( \frac{1}{r}\frac{\partial\psi}{\partial\theta} \right) \right]$,

$\frac{\partial^{2}\psi}{\partial y^{2}}=\sin^{2} \theta\frac{\partial^{2}\psi}{\partial r^{2}}+\cos^{2} \theta\left( \frac{1}{r}\frac{\partial\psi}{\partial r}+\frac{1}{r^{2}}\frac{\partial^{2}\psi}{\partial\theta^{2}} \right)+2\sin\theta\cos\theta\left[ \frac{\partial}{\partial r}\left( \frac{1}{r}\frac{\partial\psi}{\partial\theta} \right) \right]$,

$\frac{\partial^{2}\psi}{\partial x\partial y}=\sin\theta\cos\theta\left[ \frac{\partial^{2}\psi}{\partial r^{2}}-\left( \frac{1}{r}\frac{\partial\psi}{\partial r}+\frac{1}{r^{2}}\frac{\partial^{2}\psi}{\partial\theta^{2}} \right) \right]+\left( \cos^{2} \theta-\sin^{2} \theta\right)\left[ \frac{\partial}{\partial r}\left( \frac{1}{r}\frac{\partial\psi}{\partial\theta} \right) \right]$.

Adding the second derivative can get the following equation.

$\frac{\partial^{2}\psi}{\partial x^{2}}+\frac{\partial^{2}\psi}{\partial y^{2}}=\frac{\partial^{2}\psi}{\partial r^{2}}+\frac{1}{r}\frac{\partial\psi}{\partial r}+\frac{1}{r^{2}}\frac{\partial^{2}\psi}{\partial\theta^{2}}$ (A155)

So the stress compatibility equation in the polar coordinate system can be expressed as the following equation.

$\left( \frac{\partial^{2}}{\partial r^{2}}+\frac{1}{r}\frac{\partial}{\partial r}+\frac{1}{r^{2}}\frac{\partial^{2}}{\partial\theta^{2}} \right)^{2}\psi=0$  (A156)

Because,

$\frac{\partial}{\partial r}\left( \frac{1}{r}\frac{\partial\psi}{\partial r} \right)=\frac{-1}{r^{2}}\frac{\partial\psi}{\partial r}+\frac{1}{r}\frac{\partial^{2}\psi}{\partial r^{2}}$,

$\frac{\partial^{2}}{\partial r^{2}}\left( \frac{1}{r}\frac{\partial\psi}{\partial r} \right)=\frac{\partial}{\partial r}\left( \frac{-1}{r^{2}}\frac{\partial\psi}{\partial r} \right)+\frac{\partial}{\partial r}\left( \frac{1}{r}\frac{\partial^{2}\psi}{\partial r^{2}} \right)=\frac{2}{r^{3}}\frac{\partial\psi}{\partial r}+\frac{-1}{r^{2}}\frac{\partial^{2}\psi}{\partial r^{2}}+\frac{-1}{r^{2}}\frac{\partial^{2}\psi}{\partial r^{2}}+\frac{1}{r}\frac{\partial^{3}\psi}{\partial r^{3}}$,

$\frac{\partial}{\partial r}\left( \frac{1}{r^{2}}\frac{\partial^{2}\psi}{\partial\theta^{2}} \right)=\frac{-2}{r^{3}}\frac{\partial^{2}\psi}{\partial\theta^{2}}+\frac{1}{r^{2}}\frac{\partial^{3}\psi}{\partial\theta^{2}\partial r}$,

$\frac{\partial^{2}}{\partial r^{2}}\left( \frac{1}{r^{2}}\frac{\partial^{2}\psi}{\partial\theta^{2}} \right)=\frac{\partial}{\partial r}\left( \frac{-2}{r^{3}}\frac{\partial^{2}\psi}{\partial\theta^{2}}+\frac{1}{r^{2}}\frac{\partial^{3}\psi}{\partial\theta^{2}\partial r} \right)=\frac{6}{r^{4}}\frac{\partial^{2}\psi}{\partial\theta^{2}}+\frac{-2}{r^{3}}\frac{\partial^{2}\psi}{\partial\theta^{2}\partial r}+\frac{-2}{r^{3}}\frac{\partial^{3}\psi}{\partial\theta^{2}\partial r}+\frac{1}{r^{2}}\frac{\partial^{4}\psi}{\partial\theta^{2}\partial^{2}r}$,

$\frac{\partial^{2}}{\partial\theta^{2}}\left( \frac{1}{r}\frac{\partial\psi}{\partial r} \right)=\frac{1}{r}\frac{\partial^{3}\psi}{\partial r\partial\theta^{2}}$,

$\frac{\partial}{\partial\theta}\left( \frac{1}{r^{2}}\frac{\partial^{2}\psi}{\partial\theta^{2}} \right)=\frac{1}{r^{2}}\frac{\partial^{3}\psi}{\partial\theta^{3}}$,

$\frac{\partial^{2}}{\partial\theta^{2}}\left( \frac{1}{r^{2}}\frac{\partial^{2}\psi}{\partial\theta^{2}} \right)=\frac{\partial}{\partial\theta}\left( \frac{1}{r^{2}}\frac{\partial^{3}\psi}{\partial\theta^{3}} \right)=\frac{1}{r^{2}}\frac{\partial^{4}\psi}{\partial\theta^{4}}$.

Then, the stress compatibility Eq. (A156) under non-axisymmetric conditions can be further turn into the below expression,

$\left( \frac{\partial^{2}}{\partial r^{2}}+\frac{1}{r}\frac{\partial}{\partial r}+\frac{1}{r^{2}}\frac{\partial^{2}}{\partial\theta^{2}} \right)^{2}\psi=\frac{\partial^{2}}{\partial r^{2}}\left( \frac{\partial^{2}\psi}{\partial r^{2}}+\frac{1}{r}\frac{\partial\psi}{\partial r}+\frac{1}{r^{2}}\frac{\partial^{2}\psi}{\partial\theta^{2}} \right)+\frac{1}{r}\frac{\partial}{\partial r}\left( \frac{\partial^{2}\psi}{\partial r^{2}}+\frac{1}{r}\frac{\partial\psi}{\partial r}+\frac{1}{r^{2}}\frac{\partial^{2}\psi}{\partial\theta^{2}} \right)+\frac{1}{r^{2}}\frac{\partial^{2}}{\partial\theta^{2}}\left( \frac{\partial^{2}\psi}{\partial r^{2}}+\frac{1}{r}\frac{\partial\psi}{\partial r}+\frac{1}{r^{2}}\frac{\partial^{2}\psi}{\partial\theta^{2}} \right)=\frac{\partial^{4}\psi}{\partial r^{4}}+\frac{2}{r}\frac{\partial^{3}\psi}{\partial r^{3}}+\frac{-1}{r^{2}}\frac{\partial^{2}\psi}{\partial r^{2}}+\frac{1}{r^{3}}\frac{\partial\psi}{\partial r}+\frac{1}{r^{4}}\frac{\partial^{4}\psi}{\partial\theta^{4}}+\frac{4}{r^{4}}\frac{\partial^{2}\psi}{\partial\theta^{2}}+\frac{2}{r^{2}}\frac{\partial^{4}\psi}{\partial\theta^{2}\partial^{2}r}+\frac{-2}{r^{3}}\frac{\partial^{3}\psi}{\partial\theta^{2}\partial r}$ (A157)

The stress state of one point is usually called the set of stresses on all oblique cross-sections at the point. In the plane stress state, any oblique cross-section is defined by the angle $\alpha$ between its normal $n$ and the positive horizontal coordinate axis $x$, as shown in Fig. s1. Among them, the angle α is defined to be positive when rotating counterclockwise from the x-axis.


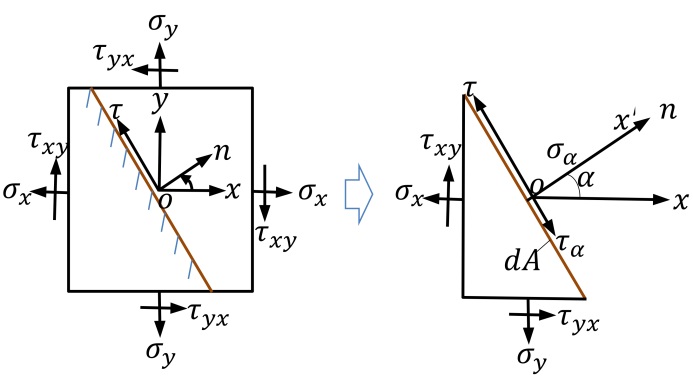


**Fig. s1.** Stress state of one point.

According to the force balance condition, $\sum F_{n}=0$, $\sum F_{t}=0$, the expressions of normal stress and shear stress on any oblique cross-sections can be obtained.

$\sigma_{\alpha}=\frac{\sigma_{x}+\sigma_{y}}{2}+\frac{\sigma_{x}-\sigma_{y}}{2}\cos2\alpha-\tau_{xy}\sin2\alpha$ (A158)

$\tau_{\alpha}=\frac{\sigma_{x}-\sigma_{y}}{2}\sin2\alpha+\tau_{xy}\cos2\alpha$ (A159)

The normal stress and shear stress expressions on any oblique cross-sections of the element body can be converted and superimposed to obtain the circle equation. This circle is called the stress circle or Mohr circle.

$\left( \sigma_{x^{'}}-\frac{\sigma_{x}+\sigma_{y}}{2} \right)^{2}+\tau_{x^{'}y^{'}}^{2}=\left( \sqrt{\left( \frac{\sigma_{x}-\sigma_{y}}{2} \right)^{2}+\tau_{xy}^{2}} \right)^{2}$ (A160)

One of the characteristics of the stress circle is the double angle correspondence. That is, the rotation angle of the radius on the stress circle is equal to twice the normal rotation angle of the oblique section on the corresponding element body, as shown in Fig. s2.


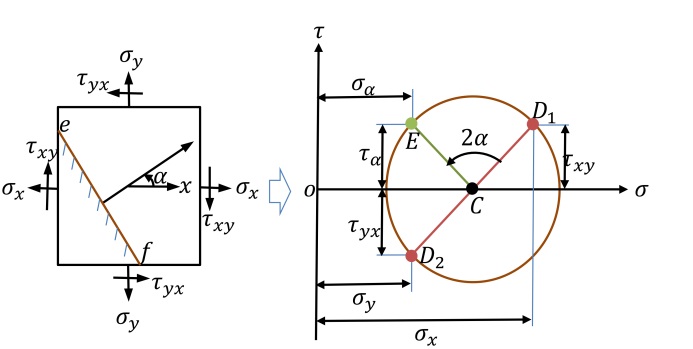


**Fig. s2.** Characteristics of the stress circle.

Therefore, in order to solving the stress compatibility Eq. (A157), constructing the following equation,

$\psi\left( r,\theta\right)=f_{1}\left( r \right)+f_{2}\left( r \right)\cos2\theta$ (A161)

Because,

$\frac{\partial\psi}{\partial r}=\frac{df_{1}\left( r \right)}{dr}+\frac{df_{2}\left( r \right)}{dr}\cos2\theta$, $\frac{\partial^{2}\psi}{\partial r^{2}}=\frac{d^{2}f_{1}\left( r \right)}{dr^{2}}+\frac{d^{2}f_{2}\left( r \right)}{dr^{2}}\cos2\theta$,

$\frac{\partial^{3}\psi}{\partial r^{3}}=\frac{d^{3}f_{1}\left( r \right)}{dr^{3}}+\frac{d^{3}f_{2}\left( r \right)}{dr^{3}}\cos2\theta$, $\frac{\partial^{4}\psi}{\partial r^{4}}=\frac{d^{4}f_{1}\left( r \right)}{dr^{4}}+\frac{d^{4}f_{2}\left( r \right)}{dr^{4}}\cos2\theta$,

$\frac{\partial\psi}{\partial\theta}={-2f}_{2}\left( r \right)\sin2\theta$, $\frac{\partial^{2}\psi}{\partial\theta^{2}}={-4f}_{2}\left( r \right)\cos2\theta$, $\frac{\partial^{3}\psi}{\partial\theta^{3}}={8f}_{2}\left( r \right)\sin2\theta$, $\frac{\partial^{4}\psi}{\partial\theta^{4}}={16f}_{2}\left( r \right)\cos2\theta$,

$\frac{\partial^{4}\psi}{\partial r^{2}\partial\theta^{2}}=\frac{\partial^{2}}{\partial\theta^{2}}\left( \frac{d^{2}f_{2}\left( r \right)}{dr^{2}}\cos2\theta\right)=\frac{\partial}{\partial\theta}\left( \frac{d^{2}f_{2}\left( r \right)}{dr^{2}}\left( -2\sin2\theta\right) \right)=-4\cos2\theta\frac{d^{2}f_{2}\left( r \right)}{dr^{2}}$,

$\frac{\partial^{3}\psi}{\partial\theta^{2}\partial r}=\frac{\partial}{\partial r}\frac{\partial^{2}\psi}{\partial\theta^{2}}=\frac{\partial}{\partial r}\left( {-4f}_{2}\left( r \right)\cos2\theta\right)=-4\cos2\theta\frac{df_{2}\left( r \right)}{dr}$.

Then, the stress compatibility Eq. (A157) can be further turn into the below expression,

$\left( \frac{\partial^{2}}{\partial r^{2}}+\frac{1}{r}\frac{\partial}{\partial r}+\frac{1}{r^{2}}\frac{\partial^{2}}{\partial\theta^{2}} \right)^{2}\psi=\frac{d^{4}f_{1}\left( r \right)}{dr^{4}}+\frac{2}{r}\frac{d^{3}f_{1}\left( r \right)}{dr^{3}}+\frac{-1}{r^{2}}\frac{d^{2}f_{1}\left( r \right)}{dr^{2}}+\frac{1}{r^{3}}\frac{df_{1}\left( r \right)}{dr}+\cos2\theta\left( \frac{d^{4}f_{2}\left( r \right)}{dr^{4}}+\frac{2}{r}\frac{d^{3}f_{2}\left( r \right)}{dr^{3}}+\frac{-9}{r^{2}}\frac{d^{2}f_{2}\left( r \right)}{dr^{2}}+\frac{9}{r^{3}}\frac{df_{2}\left( r \right)}{dr} \right)$ (A162)

Introducing an intermediate variable, $r=e^{t}$, then

$\frac{df_{1}\left( r \right)}{dr}=\frac{df_{1}\left( r \right)}{dt}\frac{dt}{dr}=\frac{df_{1}\left( r \right)}{dt}\frac{1}{r}$,

$\frac{d^{2}f_{1}\left( r \right)}{dr^{2}}=\frac{d\left[ \frac{df_{1}\left( r \right)}{dt}\frac{1}{r} \right]}{dr}=-\frac{1}{r^{2}}\frac{df_{1}\left( r \right)}{dt}+\frac{1}{r}\frac{d\left[ \frac{df_{1}\left( r \right)}{dt} \right]}{dr}=\frac{1}{r^{2}}\left[ \frac{d^{2}f_{1}\left( r \right)}{dt^{2}}-\frac{df_{1}\left( r \right)}{dt} \right]$,

$\frac{d^{3}f_{1}\left( r \right)}{dr^{3}}=\frac{d\left\{ \frac{1}{r^{2}}\left[ \frac{d^{2}f_{1}\left( r \right)}{dt^{2}}-\frac{df_{1}\left( r \right)}{dt} \right] \right\}}{dr}=\frac{1}{r^{3}}\left[ \frac{d^{3}f_{1}\left( r \right)}{dt^{3}}-3\frac{d^{2}f_{1}\left( r \right)}{dt^{2}}+2\frac{df_{1}\left( r \right)}{dt} \right]$,

$\frac{d^{4}f_{1}\left( r \right)}{dr^{4}}=\frac{d\left\{ \frac{1}{r^{3}}\left[ \frac{d^{3}f_{1}\left( r \right)}{dt^{3}}-3\frac{d^{2}f_{1}\left( r \right)}{dt^{2}}+2\frac{df_{1}\left( r \right)}{dt} \right] \right\}}{dr}=\frac{1}{r^{4}}\left[ \frac{d^{4}f_{1}\left( r \right)}{dt^{4}}-6\frac{d^{3}f_{1}\left( r \right)}{dt^{3}}+11\frac{d^{2}f_{1}\left( r \right)}{dt^{2}}-6\frac{df_{1}\left( r \right)}{dt} \right]$.

So, the equation can be further turn into the below expression,

$\frac{d^{4}f_{1}\left( r \right)}{dr^{4}}+\frac{2}{r}\frac{d^{3}f_{1}\left( r \right)}{dr^{3}}+\frac{-1}{r^{2}}\frac{d^{2}f_{1}\left( r \right)}{dr^{2}}+\frac{1}{r^{3}}\frac{df_{1}\left( r \right)}{dr}=\frac{1}{r^{4}}\left[ \frac{d^{4}f_{1}\left( r \right)}{dt^{4}}-4\frac{d^{3}f_{1}\left( r \right)}{dt^{3}}+4\frac{d^{2}f_{1}\left( r \right)}{dt^{2}} \right]=0$ (A163)

Then, $r=e^{t}$, the equation can be further turn into,

$\frac{d^{4}f_{1}\left( t \right)}{dt^{4}}-4\frac{d^{3}f_{1}\left( t \right)}{dt^{3}}+4\frac{d^{2}f_{1}\left( t \right)}{dt^{2}}=0$ (A164)

# Appendix B. Stress and displacement component

According to the related theory of elastic mechanics, when volume force is not considered, the stress component expressed by the stress function is,

$\sigma_{r}=\frac{1}{r}\frac{\partial\psi}{\partial r}+\frac{1}{r^{2}}\frac{\partial^{2}\psi}{\partial\theta^{2}}$ (A165)

$\sigma_{\theta}=\frac{\partial^{2}\psi}{\partial r^{2}}$ (A166)

$\tau_{r\theta}=-\frac{\partial}{\partial r}\left( \frac{1}{r}\frac{\partial\psi}{\partial\theta} \right)$ (A167)

Meanwhile, the stress function can be expressed as,

$\psi\left( r,\theta\right)=A_{1}lnr+B_{1}r^{2}lnr+C_{1}r^{2}+D_{1}+\left( A_{2}r^{4}+B_{2}r^{2}+C_{2}+\frac{D_{2}}{r^{2}} \right)\cos2\theta$ (A168)

Then, there are the following relationships,

$\frac{\partial\psi}{\partial r}=\frac{A_{1}}{r}+B_{1}\left( 2rlnr+r \right)+{2C}_{1}r+\left( {4A}_{2}r^{3}+{2B}_{2}r+\frac{-2D_{2}}{r^{3}} \right)\cos2\theta$*,*

$\frac{\partial^{2}\psi}{\partial r^{2}}=\frac{{-A}_{1}}{r^{2}}+B_{1}\left( 2lnr+3 \right)+{2C}_{1}+\left( {12A}_{2}r^{2}+{2B}_{2}+\frac{6D_{2}}{r^{4}} \right)\cos2\theta$*,*

$\frac{\partial\psi}{\partial\theta}=-2\left( A_{2}r^{4}+B_{2}r^{2}+C_{2}+\frac{D_{2}}{r^{2}} \right)\sin2\theta$*,*

$\frac{\partial^{2}\psi}{\partial\theta\partial r}=-2\left( {4A}_{2}r^{3}+2B_{2}r+\frac{{-2D}_{2}}{r^{3}} \right)\sin2\theta$*,*

$\frac{\partial^{2}\psi}{\partial\theta^{2}}=-4\left( A_{2}r^{4}+B_{2}r^{2}+C_{2}+\frac{D_{2}}{r^{2}} \right)\cos2\theta$*,*

So, stress components are,

$\sigma_{r}=\left[ \frac{A_{1}}{r^{2}}+B_{1}\left( 2lnr+1 \right)+{2C}_{1} \right]+\left( \frac{-6D_{2}}{r^{4}}-2B_{2}-4\frac{C_{2}}{r^{2}} \right)\cos2\theta$ (A169)

$\sigma_{\theta}=\left[ \frac{{-A}_{1}}{r^{2}}+B_{1}\left( 2lnr+3 \right)+{2C}_{1} \right]+\left( {12A}_{2}r^{2}+{2B}_{2}+\frac{6D_{2}}{r^{4}} \right)\cos2\theta$ (A170)

$\tau_{r\theta}=2\left( {3A}_{2}r^{2}+B_{2}+\frac{{-3D}_{2}}{r^{4}}-\frac{C_{2}}{r^{2}} \right)\sin2\theta$ (A171)

Strain component can be obtained by bringing the stress component into the physical equations.

$\varepsilon_{r}=\frac{1}{E}\left( \sigma_{r}-\mu\sigma_{\theta} \right)=\frac{1}{E}\left\{ \left[ \frac{A_{1}}{r^{2}}+B_{1}\left( 2lnr+1 \right)+{2C}_{1} \right]+\left[ \frac{-6D_{2}}{r^{4}}-2B_{2}-4\frac{C_{2}}{r^{2}} \right]\cos2\theta-\mu\left[ \left( \frac{{-A}_{1}}{r^{2}}+B_{1}\left( 2lnr+3 \right)+{2C}_{1} \right)+\left( {12A}_{2}r^{2}+{2B}_{2}+\frac{6D_{2}}{r^{4}} \right)\cos2\theta\right] \right\}$ (A172)

$\varepsilon_{\theta}=\frac{1}{E}\left( \sigma_{\theta}-\mu\sigma_{r} \right)=\frac{1}{E}\left\{ \left[ \left( \frac{{-A}_{1}}{r^{2}}+B_{1}\left( 2lnr+3 \right)+{2C}_{1} \right)+\left( {12A}_{2}r^{2}+{2B}_{2}+\frac{6D_{2}}{r^{4}} \right)\cos2\theta\right]-\mu\left[ \left[ \frac{A_{1}}{r^{2}}+B_{1}\left( 2lnr+1 \right)+{2C}_{1} \right]+\left[ \frac{-6D_{2}}{r^{4}}-2B_{2}-4\frac{C_{2}}{r^{2}} \right]\cos2\theta\right] \right\}$ (A173)

$\gamma_{r\theta}=\frac{2\left( 1+\mu\right)}{E}\tau_{r\theta}=\frac{4\left( 1+\mu\right)}{E}\left( {3A}_{2}r^{2}+B_{2}+\frac{{-3D}_{2}}{r^{4}}-\frac{C_{2}}{r^{2}} \right)\sin2\theta$ (A174)

Displacement component can be obtained by bring the strain component into the geometric equations and integrating them.

Geometric equations are,

$\varepsilon_{r}=\frac{\partial u_{r}}{\partial r}$ (A175)

$\varepsilon_{\theta}=\frac{u_{r}}{r}+\frac{1}{r}\frac{\partial u_{\theta}}{\partial\theta}$ (A176)

$\gamma_{r\theta}=\frac{1}{r}\frac{\partial u_{r}}{\partial\theta}+\frac{{\partial u}_{\theta}}{\partial r}-\frac{u_{\theta}}{r}$ (A177)

Displacement components are,

$\frac{\partial u_{r}}{\partial r} E=\left[ \frac{A_{1}}{r^{2}}\left( 1+\mu\right)+B_{1}\left( 2lnr+1-2lnr\mu-3\mu\right)+{2C}_{1}\left( 1-\mu\right) \right]-\left[ \frac{6D_{2}}{r^{4}}\left( 1+\mu\right)+2B_{2}\left( 1+\mu\right)+4\frac{C_{2}}{r^{2}}{+12A}_{2}r^{2}\mu\right]\cos2\theta$ (A178)

$u_{r}E=\left\{ -\frac{A_{1}}{r}\left( 1+\mu\right)+B_{1}\left[ 2r\left( lnr-1 \right)+r-2\mu r\left( lnr-1 \right)-3\mu r \right]+{2C}_{1}\left( 1-\mu\right)r \right\}+\left[ \frac{2D_{2}}{r^{3}}\left( 1+\mu\right)-2B_{2}\left( 1+\mu\right)r+4\frac{C_{2}}{r}{-4A}_{2}r^{3}\mu\right]\cos2\theta+M\left( r \right)$ (A179)

$\frac{\partial u_{\theta}}{\partial\theta}E=\left[ B_{1}r\left( 2lnr+6-4\mu lnr-2lnr-2\mu\right) \right]+\left[ \frac{4D_{2}}{r^{3}}\left( 1+\mu\right)+{4B}_{2}r\left( 1+\mu\right){+4A}_{2}r^{3}\left( \mu+3 \right)+4\frac{C_{2}}{r}\left( \mu-1 \right) \right]\cos2\theta-M\left( r \right)$ (A180)

$u_{\theta} E=\left[ {2B}_{1}r\left( 3-2\mu lnr-\mu\right) \right]\theta+\left[ 2\left( 1+\mu\right)\frac{D_{2}}{r^{3}}+{2B}_{2}\left( 1+\mu\right)r+{\left( 6+2\mu\right)A}_{2}r^{3}+2\frac{C_{2}}{r}\left( \mu-1 \right) \right]\sin2\theta-\int M\left( r \right)d\theta+N\left( \theta\right)$ (A181)

According to the single value condition of the displacement, $\left( u_{\theta} \right)_{\theta=2\pi+\theta^{'}}=\left( u_{\theta} \right)_{\theta=\theta^{'}}$ , then $B_{1}=0$, and the symmetry of the displacement, $\left( u_{\theta} \right)_{\theta=0}=\left( u_{\theta} \right)_{\theta=\pi}$, then $N\left( \theta\right)=0$，$M\left( r \right)=0$, the stress component and displacement component can be simplified as Eqs. (10~14).
